# Supplementary material for: In silico predictions of protein interactions between Zika virus and human host
Source: PeerJ. 2021 Aug 24;9:e11770. doi: 10.7717/peerj.11770 (PMC8395582; doi:10.7717/peerj.11770)
Supplement: Supplemental Information 4 [file peerj-09-11770-s004.pdf]

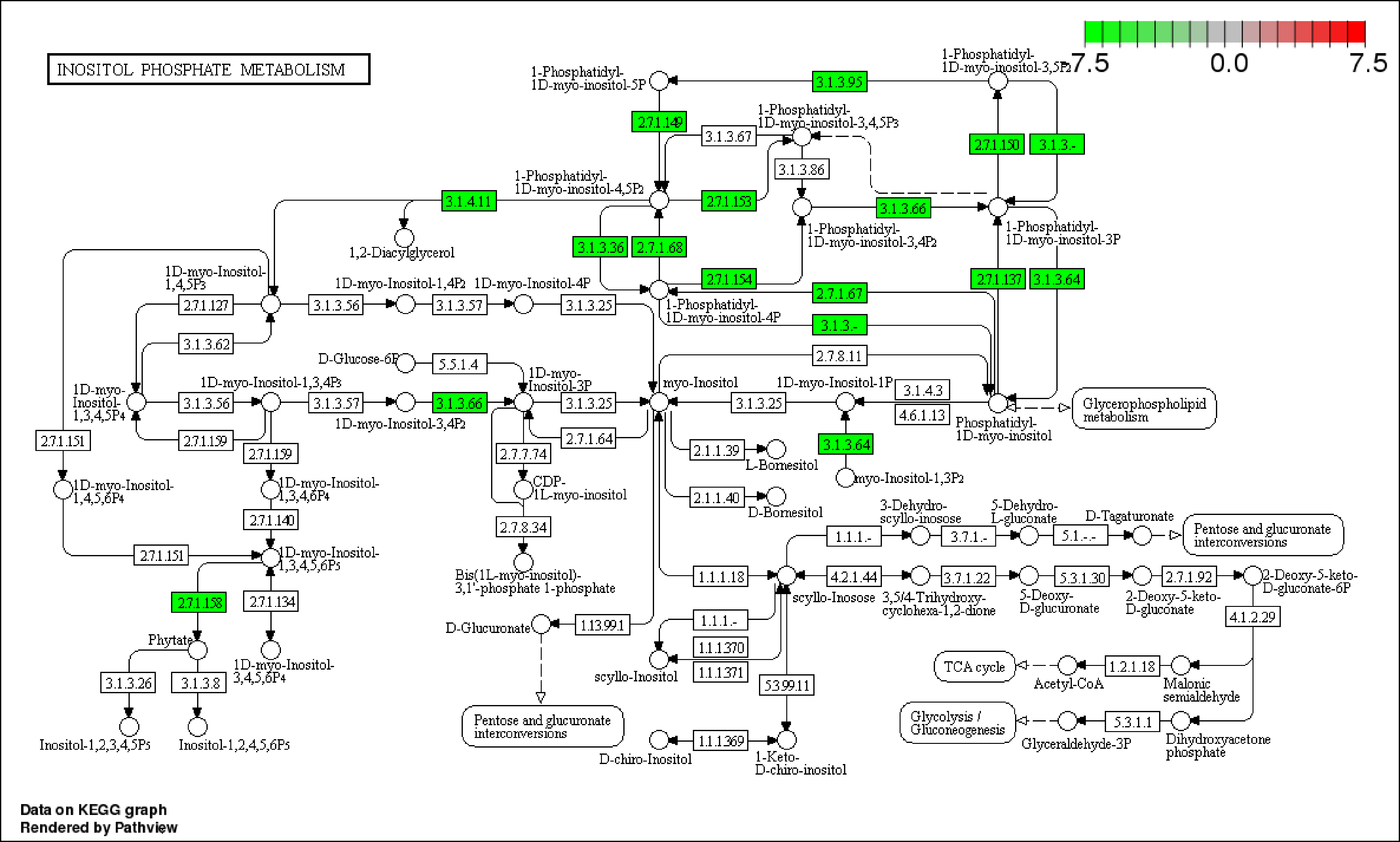

INOSITOL PHOSPHATE METABOLISM
-7.5
0.0
7.5
1-Phosphatidyl-1D-myo-inositol-5P
3.1.3.95
1-Phosphatidyl-1D-myo-inositol-3,5P2
2.7.1.149
3.1.3.67
1-Phosphatidyl-1D-myo-inositol-3,4,5P3
2.7.1.150
3.1.3.-
3.1.3.86
1-Phosphatidyl-1D-myo-inositol-4,5P2
3.1.4.11
2.7.1.153
3.1.3.66
1-Phosphatidyl-1D-myo-inositol-3P
1-Phosphatidyl-1D-myo-inositol-3,4P2
1,2-Diacylglycerol
3.1.3.36
2.7.1.68
2.7.1.154
2.7.1.137
3.1.3.64
1D-myo-Inositol-1,4,5P3
1D-myo-Inositol-1,4P2
1D-myo-Inositol-4P
2.7.1.67
1-Phosphatidyl-1D-myo-inositol-4P
2.7.1.127
3.1.3.56
3.1.3.57
3.1.3.25
3.1.3.-
3.1.3.62
2.7.8.11
D-Glucose-6P
5.5.1.4
1D-myo-Inositol-3P
myo-Inositol
1D-myo-Inositol-1P
3.1.4.3
4.6.1.13
Glycerophospholipid metabolism
1D-myo-Inositol-1,3,4,5P4
1D-myo-Inositol-1,3,4P3
3.1.3.56
3.1.3.57
3.1.3.66
3.1.3.25
Phosphatidyl-1D-myo-inositol
1D-myo-Inositol-3,4P2
2.7.1.159
2.7.1.64
3.1.3.64
2.7.1.151
2.7.1.159
2.7.7.74
2.1.1.39
L-Bornesitol
myo-Inositol-1,3P2
1D-myo-Inositol-1,4,5,6P4
1D-myo-Inositol-1,3,4,6P4
CDP-1L-myo-inositol
2.1.1.40
D-Bornesitol
2.7.1.140
2.7.8.34
3-Dehydro-scyllo-inosose
5-Dehydro-L-gluconate
D-Tagaturonate
1.1.1.-
3.7.1.-
5.1.-.-
Pentose and glucuronate interconversions
2.7.1.151
1D-myo-Inositol-1,3,4,5,6P5
Bis(1L-myo-inositol)-3,1'-phosphate 1-phosphate
1.1.1.18
4.2.1.44
3.7.1.22
5.3.1.30
2.7.1.92
2-Deoxy-5-keto-D-gluconate-6P
scyllo-Inosose
3,5/4-Trihydroxy-cyclohexa-1,2-dione
5-Deoxy-D-glucuronate
2-Deoxy-5-keto-D-gluconate
2.7.1.158
2.7.1.134
D-Glucuronate
1.13.99.1
1.1.1.-
4.1.2.29
Phytate
1.1.1.370
1.1.1.371
TCA cycle
Acetyl-CoA
1.2.1.18
Malonic semialdehyde
1D-myo-Inositol-3,4,5,6P4
scyllo-Inositol
3.1.3.26
3.1.3.8
5.3.99.11
Glycolysis / Gluconeogenesis
5.3.1.1
Glyceraldehyde-3P
Dihydroxyacetone phosphate
Pentose and glucuronate interconversions
Inositol-1,2,3,4,5P5
Inositol-1,2,4,5,6P5
D-chiro-Inositol
1.1.1.369
1-Keto-D-chiro-inositol
Data on KEGG graph
Rendered by Pathview

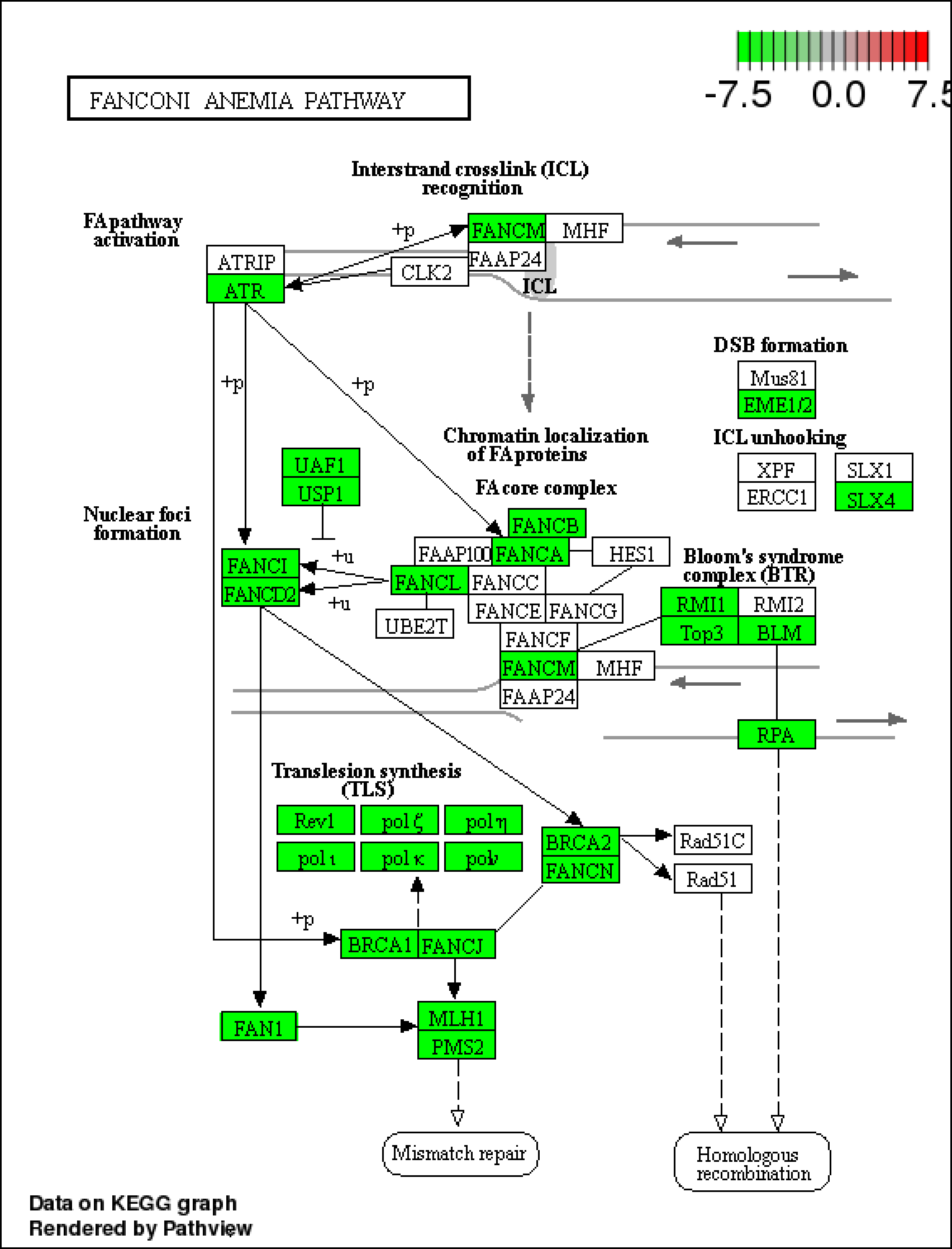

-7.5 0.0 7.5
FANCONI ANEMIA PATHWAY
Interstrand crosslink (ICL) recognition
FA pathway activation
ATRIP
ATR
+p
FANCM
MHF
FAAP24
CLK2
ICL
DSB formation
Mus81
EME1/2
ICL unhooking
XPF
ERCC1
SLX1
SLX4
Chromatin localization of FA proteins
FA core complex
UAF1
USP1
Nuclear foci formation
FANCB
FAAP100
FANCA
HES1
FANCI
FANCD2
+u
FANCL
FANCC
UBE2T
FANCE
FANCG
FANCF
Bloom's syndrome complex (BTR)
RMI1
RMI2
Top3
BLM
RPA
Translesion synthesis (TLS)
Rev1
pol ζ
pol η
pol ι
pol κ
pol ν
BRCA2
FANCN
Rad51C
Rad51
BRCA1
FANCJ
FAN1
MLH1
PMS2
Mismatch repair
Homologous recombination
Data on KEGG graph
Rendered by Pathview

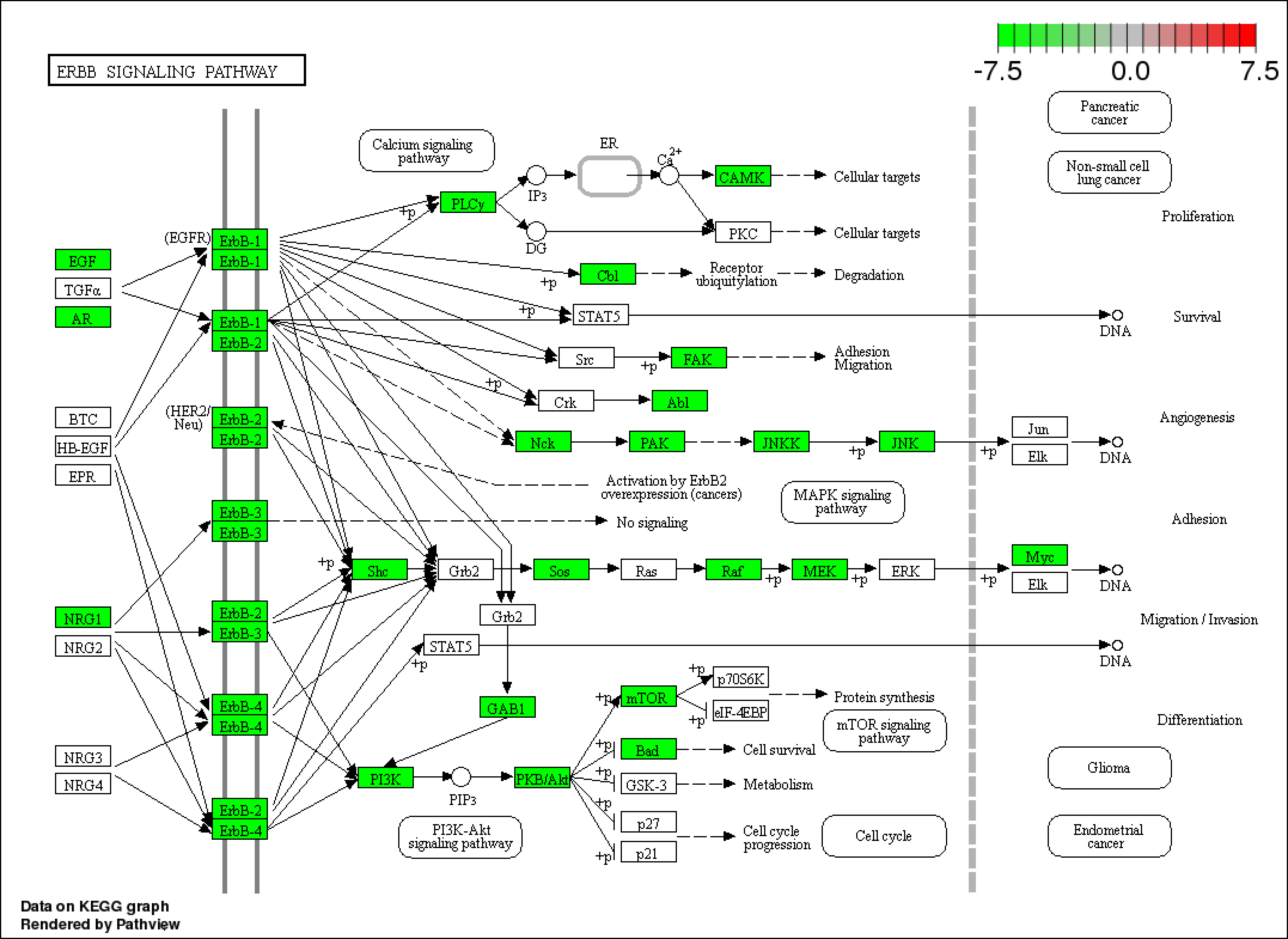
ERBB SIGNALING PATHWAY
-7.5
0.0
7.5
EGF
TGFα
AR
BTC
HB-EGF
EPR
NRG1
NRG2
NRG3
NRG4
(EGFR)
ErbB-1
ErbB-2
(HER2/Neu)
ErbB-3
ErbB-4
Calcium signaling pathway
PLCγ
IP3
DG
ER
Ca2+
CAMK
PKC
Cellular targets
Cbl
Receptor ubiquitylation
Degradation
STAT5
DNA
Src
FAK
Adhesion Migration
Crk
Abl
Nck
PAK
JNKK
JNK
Jun
Elk
Activation by ErbB2 overexpression (cancers)
MAPK signaling pathway
No signaling
Shc
Grb2
Sos
Ras
Raf
MEK
ERK
Myc
GAB1
mTOR
p70S6K
eIF-4EBP
Protein synthesis
mTOR signaling pathway
Bad
Cell survival
GSK-3
Metabolism
p27
p21
Cell cycle progression
Cell cycle
PI3K
PIP3
PKB/Akt
PI3K-Akt signaling pathway
+p
Pancreatic cancer
Non-small cell lung cancer
Proliferation
Survival
Angiogenesis
Adhesion
Migration / Invasion
Differentiation
Glioma
Endometrial cancer
Data on KEGG graph
Rendered by Pathview

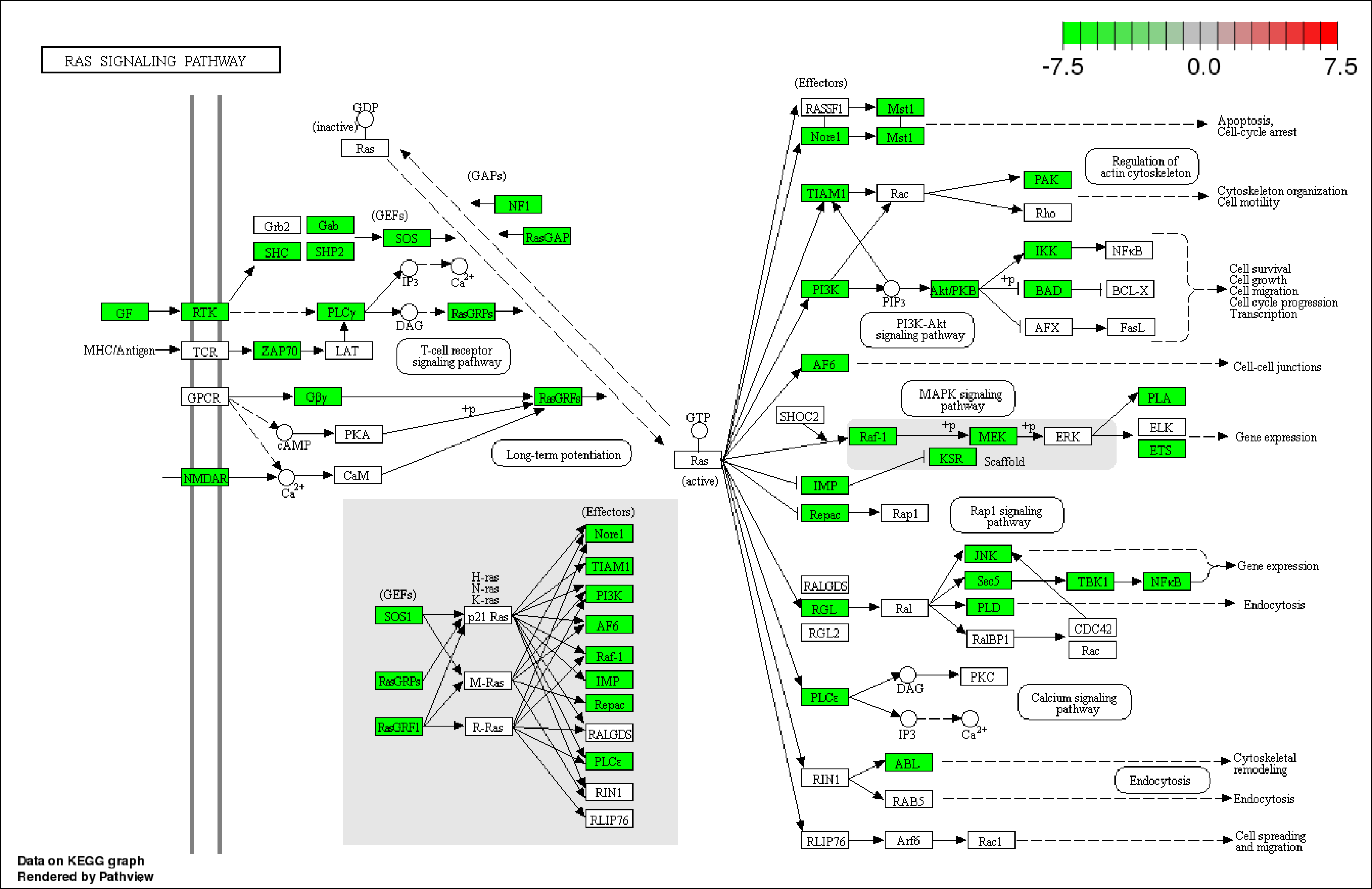

RAS SIGNALING PATHWAY
-7.5
0.0
7.5
GDP
(inactive)
Ras
(GAPs)
NF1
RasGAP
Grb2
Gab
(GEFs)
SOS
SHC
SHP2
GF
RTK
PLCγ
IP3
Ca2+
DAG
RasGRFs
MHC/Antigen
TCR
ZAP70
LAT
T-cell receptor signaling pathway
GPCR
Gβγ
RasGRFs
+p
cAMP
PKA
Long-term potentiation
NMDAR
Ca2+
CaM
GTP
Ras
(active)
(Effectors)
(GEFs)
SOS1
RasGRPs
RasGRF1
H-ras
N-ras
K-ras
p21 Ras
M-Ras
R-Ras
Nore1
TIAM1
PI3K
AF6
Raf-1
IMP
Repac
RALGDS
PLCε
RIN1
RLIP76
(Effectors)
RASSF1
Mst1
Nore1
Mst1
Apoptosis, Cell-cycle arrest
Regulation of actin cytoskeleton
TIAM1
Rac
PAK
Rho
Cytoskeleton organization Cell motility
IKK
NFκB
PI3K
PIP3
Akt/PKB
+p
BAD
BCL-X
AFX
FasL
PI3K-Akt signaling pathway
Cell survival
Cell growth
Cell migration
Cell cycle progression
Transcription
AF6
Cell-cell junctions
MAPK signaling pathway
PLA
SHOC2
Raf-1
+p
MEK
+p
ERK
ELK
ETS
Gene expression
KSR
Scaffold
IMP
Repac
Rap1
Rap1 signaling pathway
JNK
Gene expression
RALGDS
Sec5
TBK1
NFκB
RGL
Ral
PLD
Endocytosis
RGL2
RalBP1
CDC42
Rac
PLCε
DAG
PKC
Calcium signaling pathway
IP3
Ca2+
ABL
Cytoskeletal remodeling
RIN1
Endocytosis
RAB5
Endocytosis
RLIP76
Arf6
Rac1
Cell spreading and migration
Data on KEGG graph
Rendered by Pathview

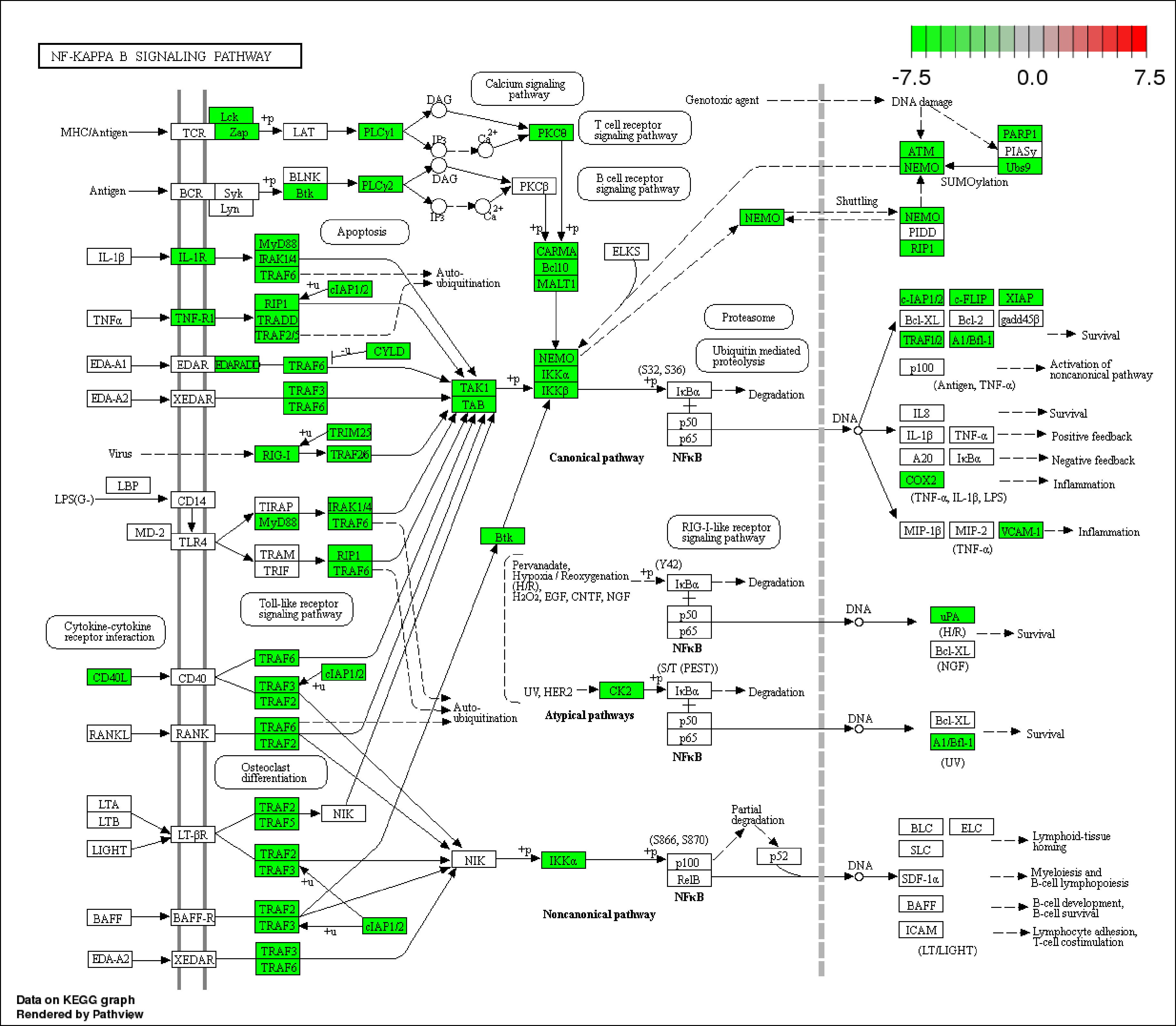
NF-KAPPA B SIGNALING PATHWAY
-7.5
0.0
7.5
Calcium signaling pathway
T cell receptor signaling pathway
B cell receptor signaling pathway
Genotoxic agent
DNA damage
MHC/Antigen
TCR
Lck
Zap
+p
LAT
PLCγ1
DAG
IP3
Ca2+
PKCθ
Antigen
BCR
Syk
Lyn
BLNK
Btk
PLCγ2
PKCβ
PARP1
PIASy
Ubs9
ATM
NEMO
SUMOylation
Shuttling
NEMO
PIDD
RIP1
Apoptosis
IL-1β
IL-1R
MyD88
IRAK1/4
TRAF6
Auto-ubiquitination
+u
cIAP1/2
TNFα
TNF-R1
RIP1
TRADD
TRAF2/5
CARMA
Bcl10
MALT1
ELKS
Proteasome
Ubiquitin mediated proteolysis
c-IAP1/2
c-FLIP
XIAP
Bcl-XL
Bcl-2
gadd45β
TRAF1/2
A1/Bfl-1
Survival
EDA-A1
EDAR
EDARADD
TRAF6
-u
CYLD
p100
Activation of noncanonical pathway
(Antigen, TNF-α)
EDA-A2
XEDAR
TRAF3
TRAF6
TAK1
TAB
NEMO
IKKα
IKKβ
(S32, S36)
IκBα
Degradation
p50
p65
DNA
IL8
Survival
IL-1β
TNF-α
Positive feedback
A20
IκBα
Negative feedback
COX2
Inflammation
(TNF-α, IL-1β, LPS)
Virus
RIG-I
TRIM25
TRAF2/6
Canonical pathway
NFκB
LBP
LPS(G-)
CD14
MD-2
TLR4
TIRAP
MyD88
IRAK1/4
TRAF6
TRAM
TRIF
RIP1
TRAF6
Btk
RIG-I-like receptor signaling pathway
MIP-1β
MIP-2
VCAM-1
Inflammation
(TNF-α)
Pervanadate, Hypoxia / Reoxygenation (H/R), H2O2, EGF, CNTF, NGF
(Y42)
IκBα
Degradation
p50
p65
NFκB
DNA
uPA
(H/R)
Survival
Bcl-XL
(NGF)
Toll-like receptor signaling pathway
Cytokine-cytokine receptor interaction
CD40L
CD40
TRAF6
cIAP1/2
TRAF3
TRAF2
UV, HER2
CK2
(S/T (PEST))
IκBα
Degradation
Atypical pathways
p50
p65
NFκB
DNA
Bcl-XL
A1/Bfl-1
Survival
(UV)
RANKL
RANK
TRAF6
TRAF2
Osteoclast differentiation
LTA
LTB
LIGHT
LT-βR
TRAF2
TRAF5
NIK
TRAF2
TRAF3
NIK
IKKα
(S866, S870)
Partial degradation
p52
p100
RelB
NFκB
DNA
Noncanonical pathway
BLC
ELC
Lymphoid-tissue homing
SLC
SDF-1α
Myeloiesis and B-cell lymphopoiesis
BAFF
BAFF-R
TRAF2
TRAF3
cIAP1/2
BAFF
B-cell development, B-cell survival
ICAM
Lymphocyte adhesion, T-cell costimulation
(LT/LIGHT)
EDA-A2
XEDAR
TRAF3
TRAF6
Data on KEGG graph
Rendered by Pathview

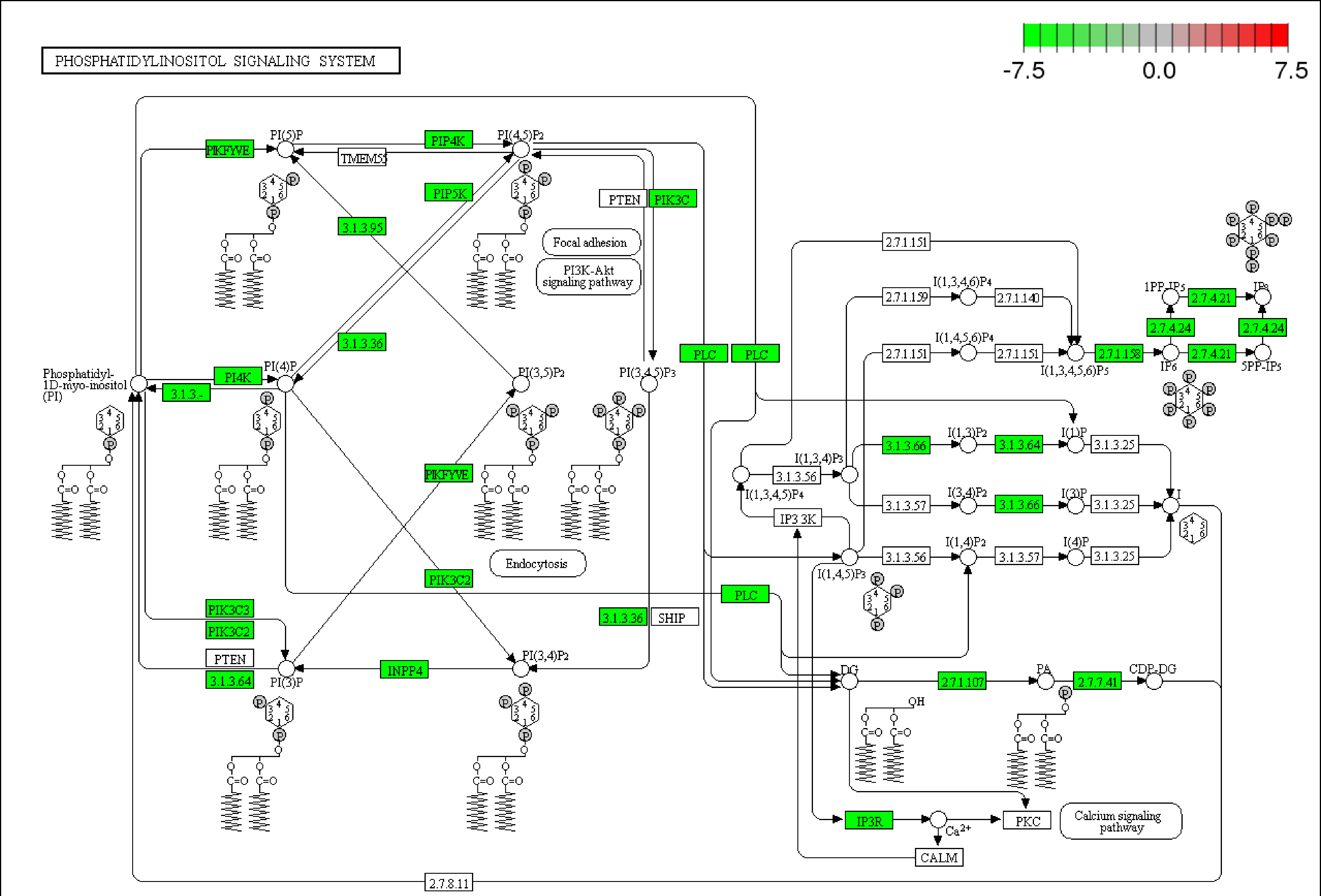

Data on KEGG graph
Rendered by Pathview

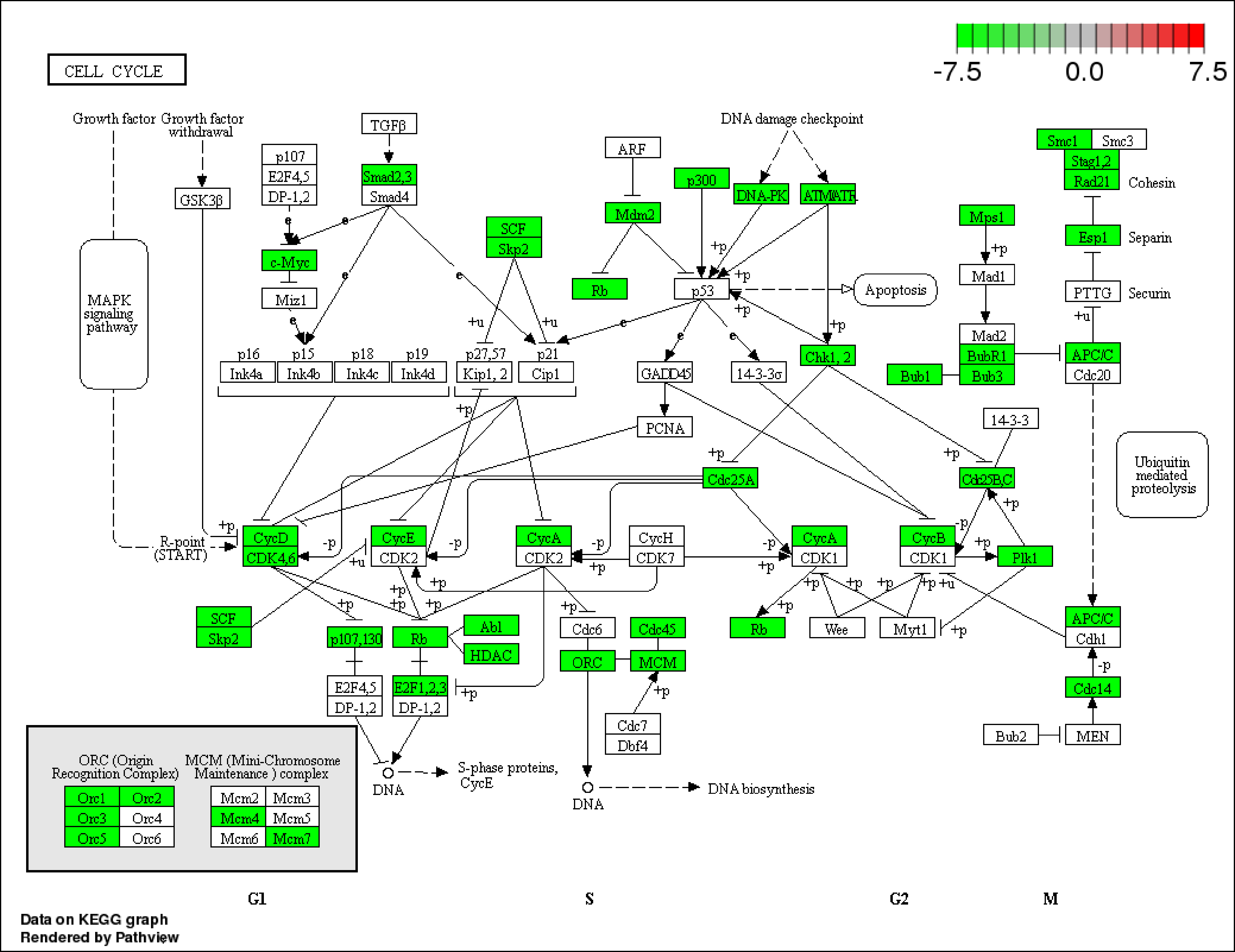

-7.5
0.0
7.5
CELL CYCLE
Growth factor
Growth factor withdrawal
GSK3β
MAPK signaling pathway
R-point (START)
p107
E2F4,5
DP-1,2
TGFβ
Smad2,3
Smad4
c-Myc
Miz1
p16
Ink4a
p15
Ink4b
p18
Ink4c
p19
Ink4d
SCF
Skp2
p27,57
Kip1, 2
p21
Cip1
ARF
Mdm2
Rb
p300
DNA damage checkpoint
DNA-PK
ATM/ATR
p53
Apoptosis
GADD45
14-3-3σ
PCNA
Chk1, 2
Cdc25A
Cdc25B,C
14-3-3
Mps1
Mad1
Mad2
BubR1
Bub1
Bub3
APC/C
Cdc20
Smc1
Smc3
Stag1,2
Rad21
Cohesin
Esp1
Separin
PTTG
Securin
Ubiquitin mediated proteolysis
CycD
CDK4,6
CycE
CDK2
CycA
CDK2
CycH
CDK7
CycA
CDK1
CycB
CDK1
Plk1
SCF
Skp2
p107,130
E2F4,5
DP-1,2
Rb
Abl
HDAC
E2F1,2,3
DP-1,2
Cdc6
Cdc45
ORC
MCM
Cdc7
Dbf4
Rb
Wee
Myt1
APC/C
Cdh1
Cdc14
Bub2
MEN
DNA
S-phase proteins, CycE
DNA
DNA biosynthesis
ORC (Origin Recognition Complex)
Orc1
Orc2
Orc3
Orc4
Orc5
Orc6
MCM (Mini-Chromosome Maintenance) complex
Mcm2
Mcm3
Mcm4
Mcm5
Mcm6
Mcm7
G1
S
G2
M
Data on KEGG graph
Rendered by Pathview

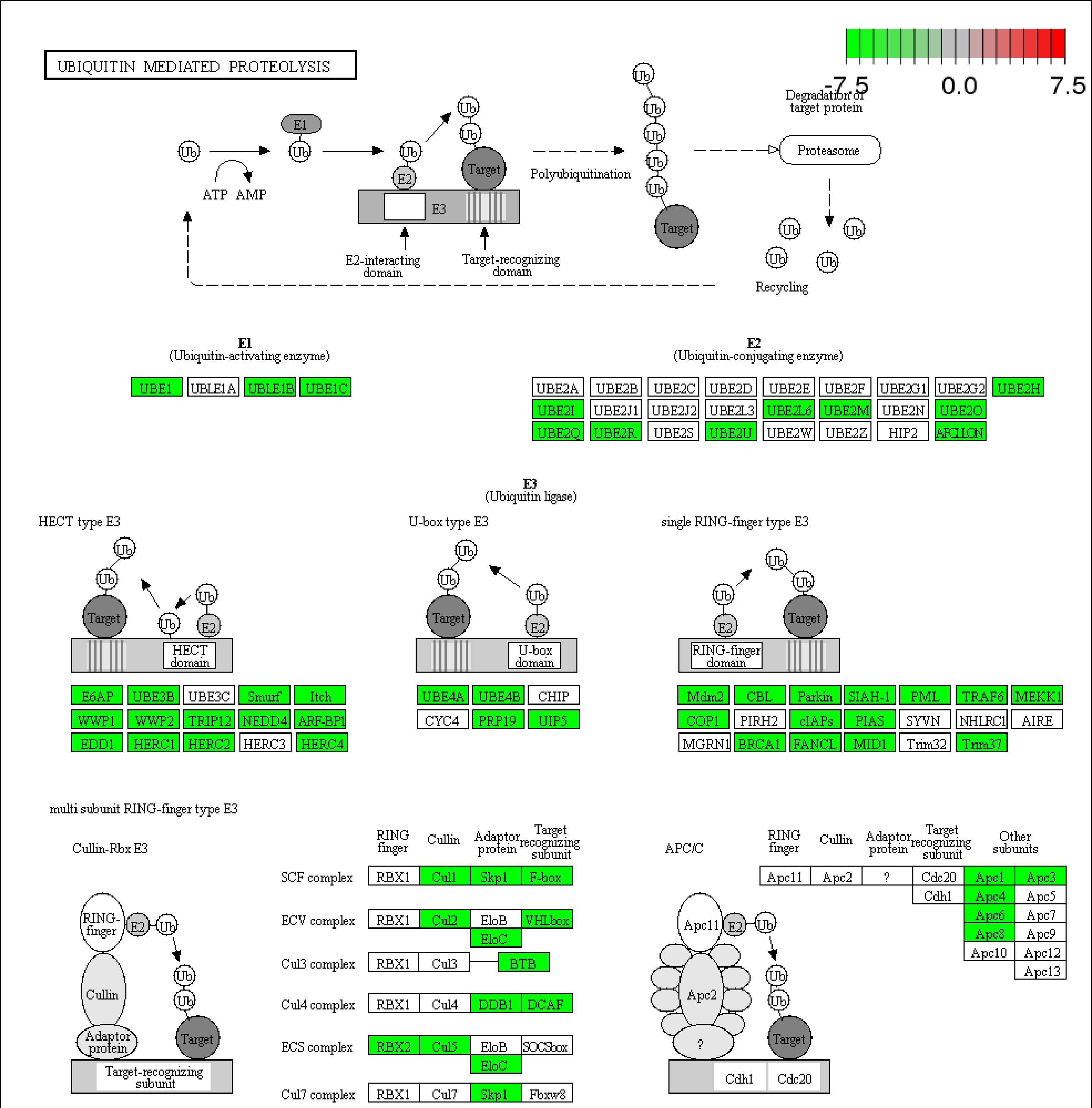

Data on KEGG graph
Rendered by Pathview

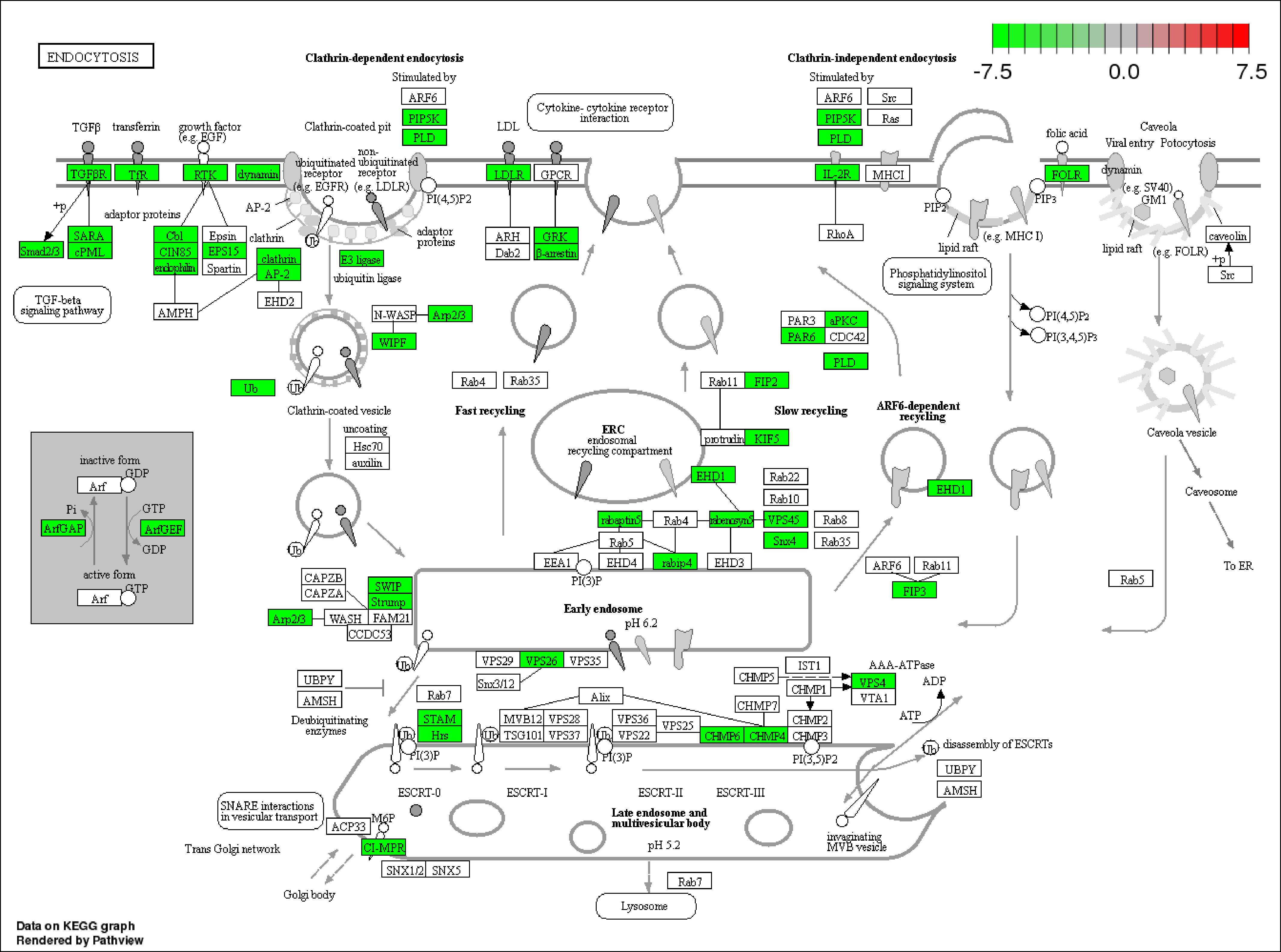
ENDOCYTOSIS
-7.5
0.0
7.5
Clathrin-dependent endocytosis
Stimulated by
ARF6
PIP5K
PLD
Clathrin-coated pit
Cytokine- cytokine receptor interaction
Clathrin-independent endocytosis
Stimulated by
ARF6
Src
PIP5K
Ras
PLD
TGFβ
transferrin
growth factor (e.g. EGF)
LDL
folic acid
Caveola
Viral entry
Potocytosis
TGFβR
TfR
RTK
dynamin
ubiquitinated receptor (e.g. EGFR)
non-ubiquitinated receptor (e.g. LDLR)
LDLR
GPCR
IL-2R
MHCI
FOLR
dynamin
(e.g. SV40)
GM1
PI(4,5)P2
AP-2
adaptor proteins
+p
adaptor proteins
clathrin
PIP2
PIP3
lipid raft
(e.g. MHC I)
lipid raft
(e.g. FOLR)
caveolin
+p
Src
Smad2/3
SARA
cPML
Cbl
CIN85
endophilin
Epsin
EPS15
Spartin
clathrin
AP-2
EHD2
E3 ligase
ubiquitin ligase
ARH
Dab2
GRK
β-arrestin
RhoA
Phosphatidylinositol signaling system
PI(4,5)P2
PI(3,4,5)P3
TGF-beta signaling pathway
AMPH
N-WASP
Arp2/3
WIPF
PAR3
aPKC
PAR6
CDC42
PLD
Ub
Ub
Clathrin-coated vesicle
Rab4
Rab35
Rab11
FIP2
Fast recycling
Slow recycling
ARF6-dependent recycling
Caveola vesicle
ERC
endosomal recycling compartment
protrudin
KIF5
uncoating
Hsc70
auxilin
EHD1
Rab22
Rab10
EHD1
Caveosome
inactive form
Arf
GDP
Pi
GTP
ArfGAP
ArfGEF
GDP
active form
Arf
GTP
Ub
rabaptin5
Rab4
rabenosyn5
VPS45
Rab8
Rab5
Snx4
Rab35
To ER
EEA1
EHD4
rabip4
EHD3
ARF6
Rab11
FIP3
Rab5
PI(3)P
CAPZB
CAPZA
SWIP
Strump
Arp2/3
WASH
FAM21
CCDC53
Early endosome
pH 6.2
Ub
VPS29
VPS26
VPS35
Snx3/12
UBPY
AMSH
Deubiquitinating enzymes
Rab7
CHMP5
IST1
CHMP1
VPS4
VTA1
AAA-ATPase
ADP
ATP
Alix
CHMP7
STAM
Hrs
MVB12
VPS28
TSG101
VPS37
VPS36
VPS22
VPS25
CHMP6
CHMP4
CHMP2
CHMP3
Ub
Ub
Ub
PI(3)P
PI(3)P
PI(3,5)P2
Ub
disassembly of ESCRTs
UBPY
AMSH
ESCRT-0
ESCRT-I
ESCRT-II
ESCRT-III
SNARE interactions in vesicular transport
ACP33
M6P
CI-MPR
Late endosome and multivesicular body
pH 5.2
invaginating MVB vesicle
Trans Golgi network
Golgi body
SNX1/2
SNX5
Rab7
Lysosome
Data on KEGG graph
Rendered by Pathview

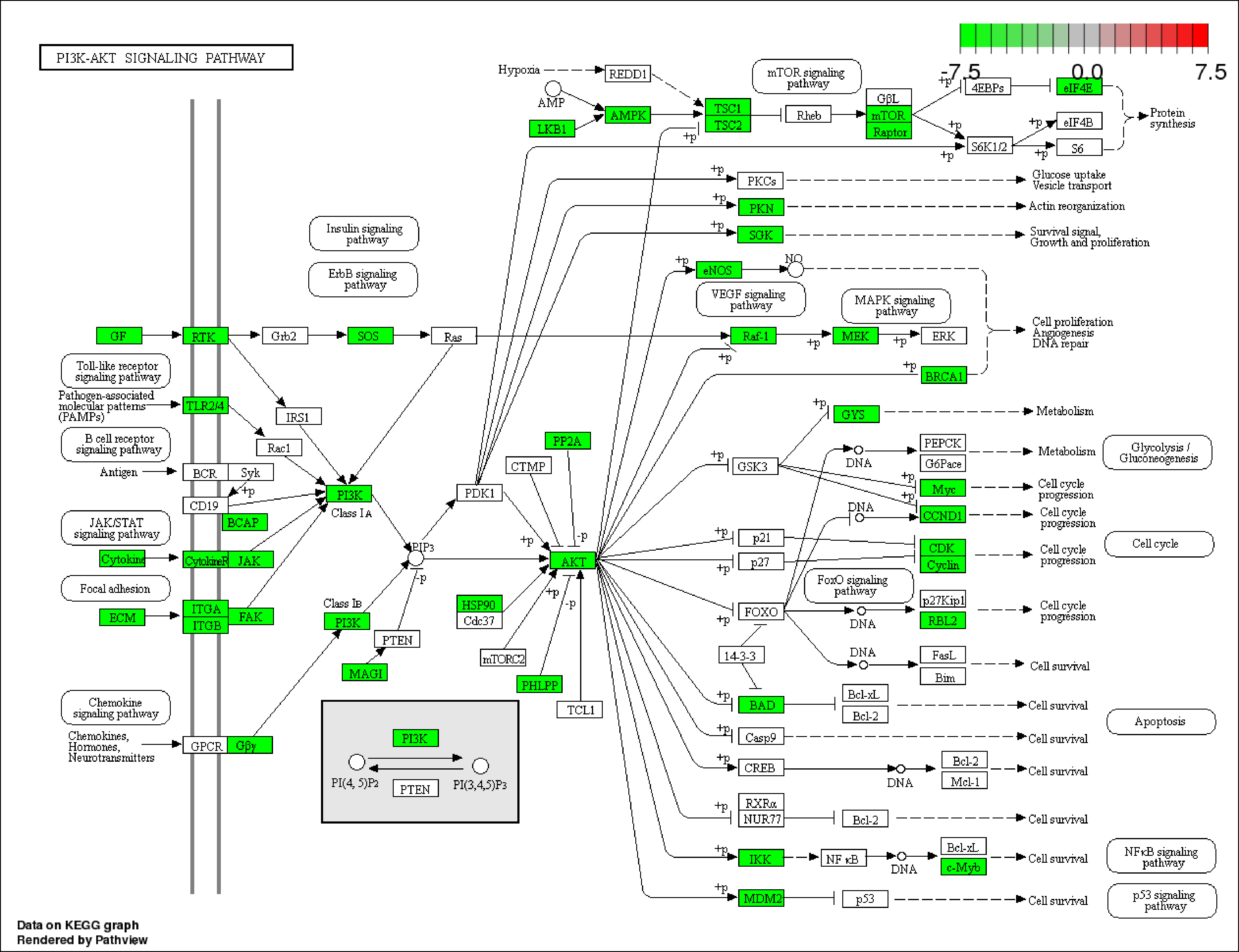
PI3K-AKT SIGNALING PATHWAY
-7.5
0.0
7.5
Hypoxia
REDD1
AMP
AMPK
LKB1
TSC1
TSC2
mTOR signaling pathway
Rheb
GβL
mTOR
Raptor
4EBPs
eIF4E
eIF4B
S6K1/2
S6
Protein synthesis
PKCs
Glucose uptake
Vesicle transport
PKN
Actin reorganization
SGK
Survival signal, Growth and proliferation
Insulin signaling pathway
ErbB signaling pathway
eNOS
NO
VEGF signaling pathway
MAPK signaling pathway
Cell proliferation
Angiogenesis
DNA repair
GF
RTK
Grb2
SOS
Ras
Raf-1
MEK
ERK
BRCA1
Toll-like receptor signaling pathway
Pathogen-associated molecular patterns (PAMPs)
TLR2/4
IRS1
Rac1
GYS
Metabolism
B cell receptor signaling pathway
Antigen
BCR
Syk
CD19
BCAP
PI3K
Class IA
PDK1
CTMP
PP2A
GSK3
DNA
PEPCK
G6Pace
Glycolysis / Gluconeogenesis
Myc
Cell cycle progression
CCND1
JAK/STAT signaling pathway
Cytokine
CytokineR
JAK
PIP3
AKT
p21
p27
CDK
Cyclin
Cell cycle
Focal adhesion
ECM
ITGA
ITGB
FAK
Class IB
HSP90
Cdc37
FoxO signaling pathway
FOXO
p27Kip1
RBL2
PTEN
MAGI
mTORC2
PHLPP
TCL1
14-3-3
FasL
Bim
Cell survival
BAD
Bcl-xL
Bcl-2
Apoptosis
Chemokine signaling pathway
Chemokines, Hormones, Neurotransmitters
GPCR
Gβγ
PI(4, 5)P2
PI(3,4,5)P3
Casp9
CREB
Mcl-1
RXRα
NUR77
IKK
NF κB
c-Myb
NFκB signaling pathway
MDM2
p53
p53 signaling pathway
+p
-p
Data on KEGG graph
Rendered by Pathview

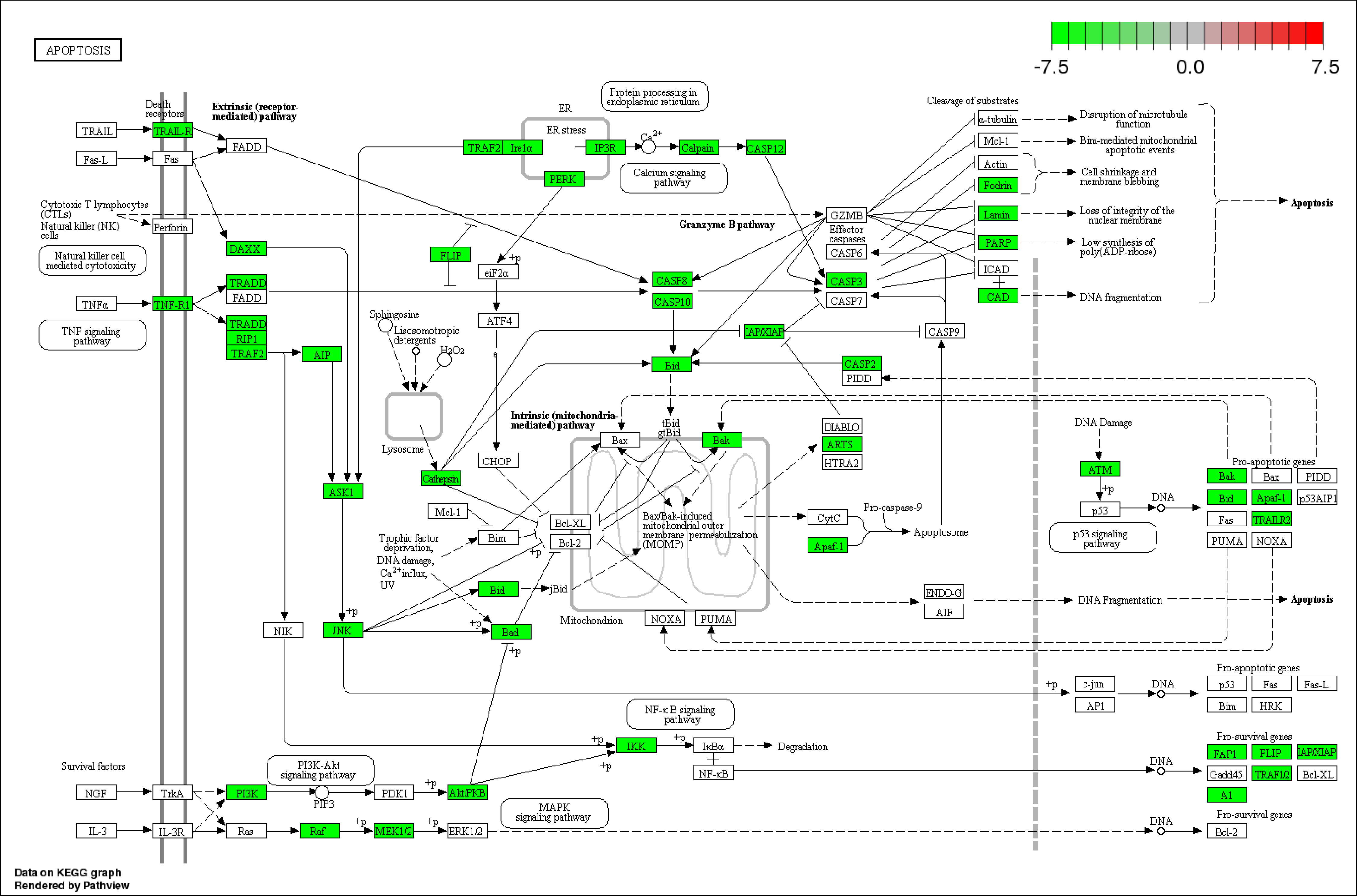
APOPTOSIS
-7.5
0.0
7.5
Death receptors
Extrinsic (receptor-mediated) pathway
TRAIL
TRAIL-R
FADD
Fas-L
Fas
Cytotoxic T lymphocytes (CTLs)
Natural killer (NK) cells
Perforin
Natural killer cell mediated cytotoxicity
DAXX
TRADD
FADD
TNFα
TNF-R1
TNF signaling pathway
TRADD
RIP1
TRAF2
AIP
ER
ER stress
TRAF2
Ire1α
PERK
IP3R
Ca2+
Calpain
CASP12
Protein processing in endoplasmic reticulum
Calcium signaling pathway
FLIP
eIF2α
ATF4
CHOP
Sphingosine
Lysosomotropic detergents
H2O2
Lysosome
Cathepsin
ASK1
Mcl-1
Bim
Trophic factor deprivation, DNA damage, Ca2+ influx, UV
Bid
jBid
Bad
JNK
NIK
Granzyme B pathway
GZMB
Effector caspases
CASP6
CASP3
CASP7
CASP8
CASP10
IAP/XIAP
CASP9
Bid
CASP2
PIDD
Intrinsic (mitochondria-mediated) pathway
tBid
gtBid
Bax
Bak
Bcl-XL
Bcl-2
Bax/Bak-induced mitochondrial outer membrane permeabilization (MOMP)
Mitochondrion
NOXA
PUMA
DIABLO
ARTS
HTRA2
CytC
Apaf-1
Pro-caspase-9
Apoptosome
ENDO-G
AIF
DNA Fragmentation
Apoptosis
Cleavage of substrates
α-tubulin
Mcl-1
Actin
Fodrin
Lamin
PARP
ICAD
CAD
Disruption of microtubule function
Bim-mediated mitochondrial apoptotic events
Cell shrinkage and membrane blebbing
Loss of integrity of the nuclear membrane
Low synthesis of poly(ADP-ribose)
DNA fragmentation
Apoptosis
DNA Damage
ATM
p53
DNA
p53 signaling pathway
Pro-apoptotic genes
Bak
Bax
PIDD
Bid
Apaf-1
p53AIP1
Fas
TRAILR2
PUMA
NOXA
c-jun
AP1
DNA
Pro-apoptotic genes
p53
Fas
Fas-L
Bim
HRK
NF-κB signaling pathway
IKK
IκBα
NF-κB
Degradation
Pro-survival genes
IAP1
FLIP
IAP/XIAP
Gadd45
TRAF1/2
Bcl-XL
A1
Survival factors
NGF
TrkA
PI3K
PIP3
PDK1
Akt/PKB
PI3K-Akt signaling pathway
IL-3
IL-3R
Ras
Raf
MEK1/2
ERK1/2
MAPK signaling pathway
Pro-survival genes
Bcl-2
+p
Data on KEGG graph
Rendered by Pathview

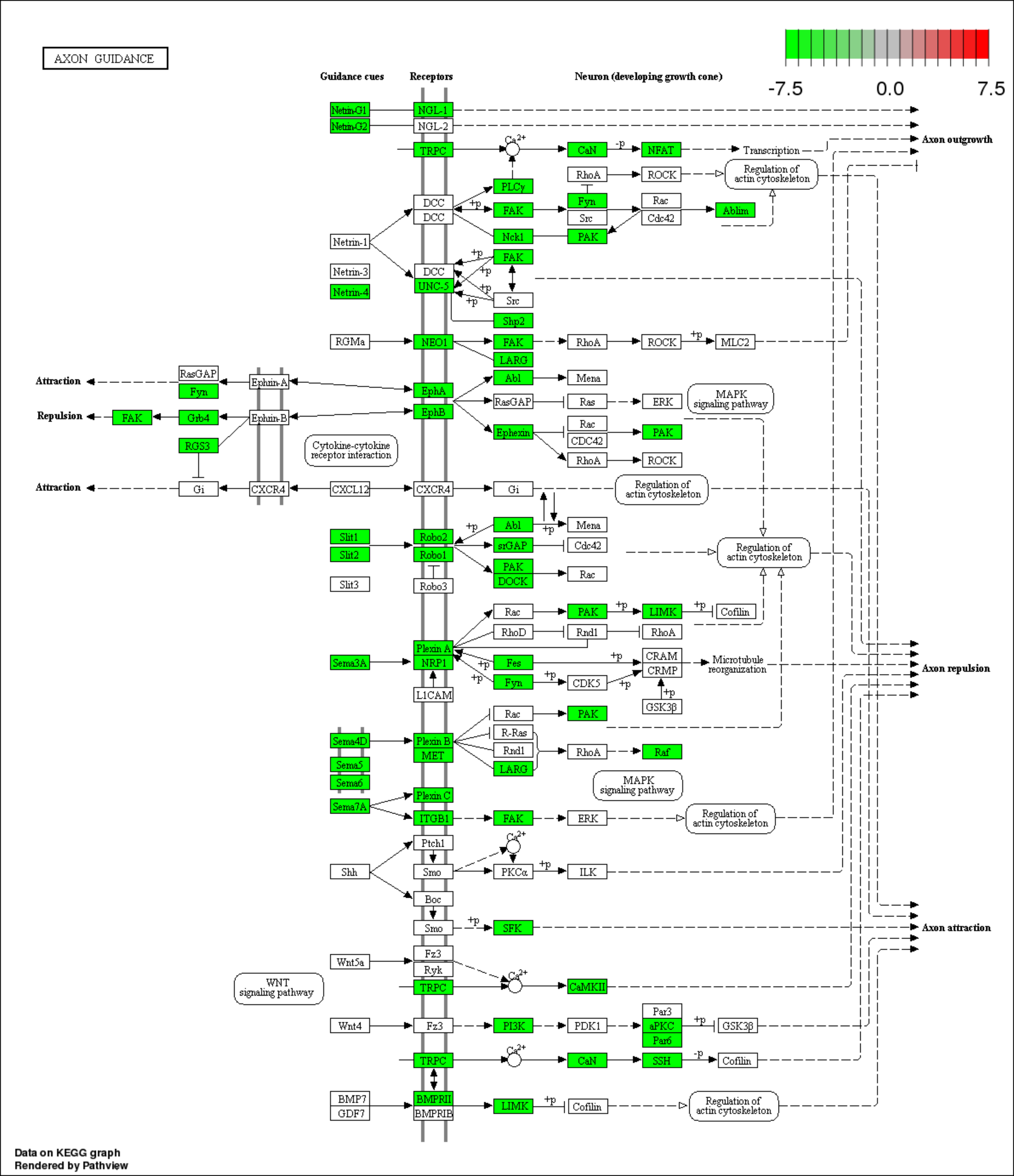
AXON GUIDANCE
-7.5
0.0
7.5
Guidance cues
Receptors
Neuron (developing growth cone)
Netrin-G1
NGL-1
Netrin-G2
NGL-2
TRPC
Ca2+
CaN
-p
NFAT
Transcription
Axon outgrowth
RhoA
ROCK
Regulation of actin cytoskeleton
PLCγ
Fyn
DCC
DCC
+p
FAK
Src
Rac
Cdc42
Ablim
Netrin-1
Nck1
PAK
FAK
Netrin-3
DCC
UNC-5
Src
Netrin-4
Shp2
RGMa
NEO1
FAK
RhoA
ROCK
MLC2
LARG
Attraction
RasGAP
Ephrin-A
EphA
Abl
Mena
Fyn
RasGAP
Ras
ERK
MAPK signaling pathway
Repulsion
FAK
Grb4
Ephrin-B
EphB
Ephexin
Rac
CDC42
PAK
RGS3
Cytokine-cytokine receptor interaction
RhoA
ROCK
Attraction
Gi
CXCR4
CXCL12
CXCR4
Gi
Regulation of actin cytoskeleton
Slit1
Robo2
Abl
Mena
Slit2
Robo1
srGAP
Cdc42
Regulation of actin cytoskeleton
PAK
DOCK
Rac
Slit3
Robo3
Rac
PAK
LIMK
Cofilin
RhoD
Rnd1
RhoA
Plexin A
Sema3A
NRP1
Fes
CRAM
CRMP
Microtubule reorganization
Axon repulsion
Fyn
CDK5
L1CAM
GSK3β
Rac
PAK
Sema4D
Plexin B
R-Ras
MET
Rnd1
RhoA
Raf
Sema5
LARG
Sema6
MAPK signaling pathway
Plexin C
Sema7A
ITGB1
FAK
ERK
Regulation of actin cytoskeleton
Ptch1
Ca2+
Shh
Smo
PKCα
ILK
Boc
Smo
SFK
Axon attraction
Fz3
Wnt5a
Ryk
WNT signaling pathway
TRPC
Ca2+
CaMKII
Par3
Wnt4
Fz3
PI3K
PDK1
aPKC
GSK3β
Par6
TRPC
Ca2+
CaN
SSH
Cofilin
BMP7
BMPRII
GDF7
BMPRIB
LIMK
Cofilin
Regulation of actin cytoskeleton
Data on KEGG graph
Rendered by Pathview

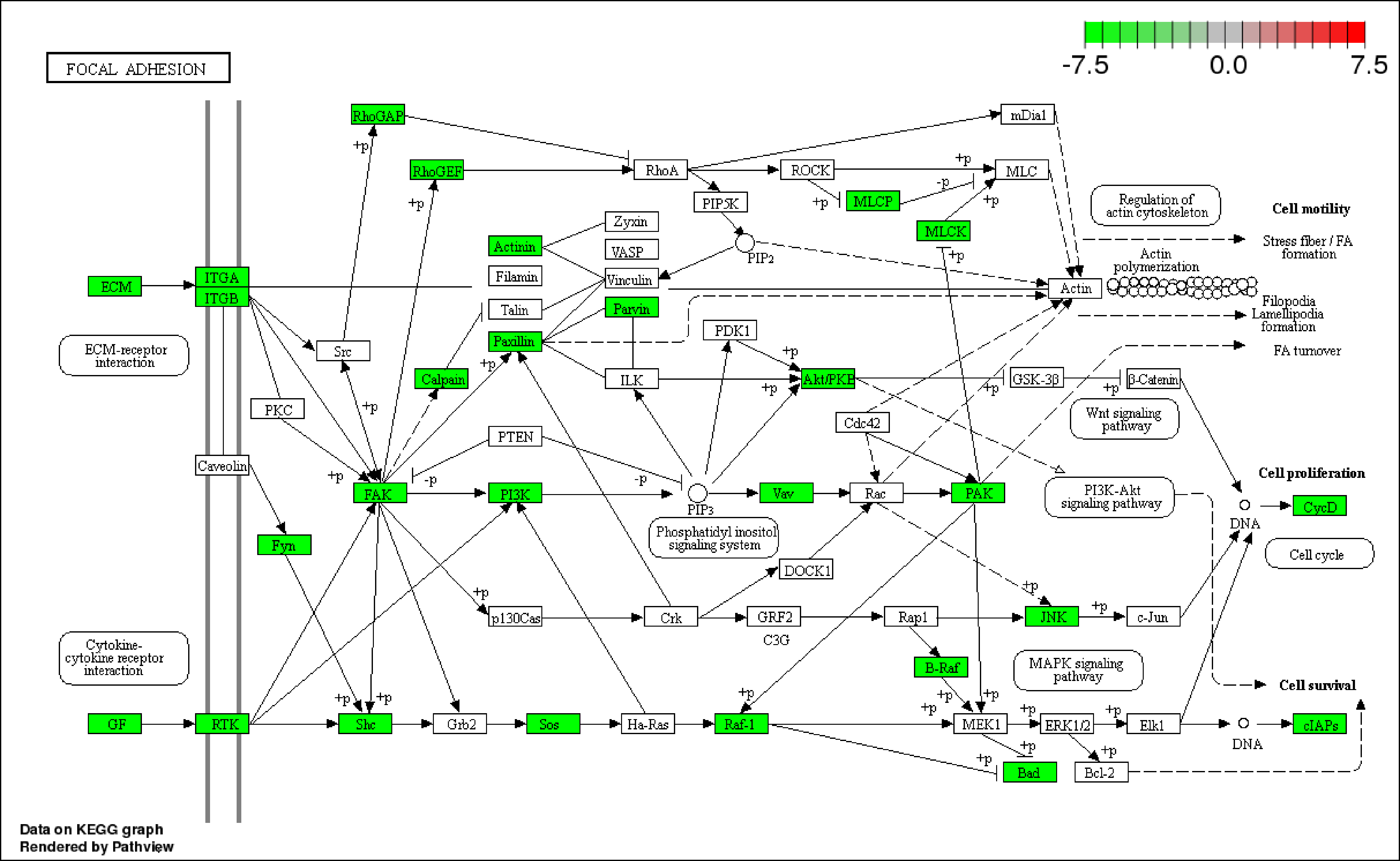

-7.5
0.0
7.5
FOCAL ADHESION
ECM
ITGA
ITGB
ECM-receptor interaction
Cytokine-cytokine receptor interaction
GF
RTK
PKC
Caveolin
Fyn
Src
RhoGAP
RhoGEF
FAK
Calpain
PI3K
Shc
Grb2
Sos
Ha-Ras
Raf-1
Actinin
Filamin
Talin
Paxillin
Zyxin
VASP
Vinculin
Parvin
ILK
PTEN
p130Cas
Crk
GRF2
C3G
DOCK1
Rap1
B-Raf
MEK1
ERK1/2
Elk1
Bad
Bcl-2
RhoA
PIP5K
PIP2
ROCK
MLCP
MLC
MLCK
mDia1
PDK1
Akt/PKB
PIP3
Phosphatidyl inositol signaling system
Vav
Rac
Cdc42
PAK
JNK
c-Jun
GSK-3β
β-Catenin
Wnt signaling pathway
PI3K-Akt signaling pathway
MAPK signaling pathway
Actin
Actin polymerization
Regulation of actin cytoskeleton
Cell motility
Stress fiber / FA formation
Filopodia Lamellipodia formation
FA turnover
Cell proliferation
DNA
CycD
Cell cycle
Cell survival
cIAPs
+p
-p
Data on KEGG graph
Rendered by Pathview

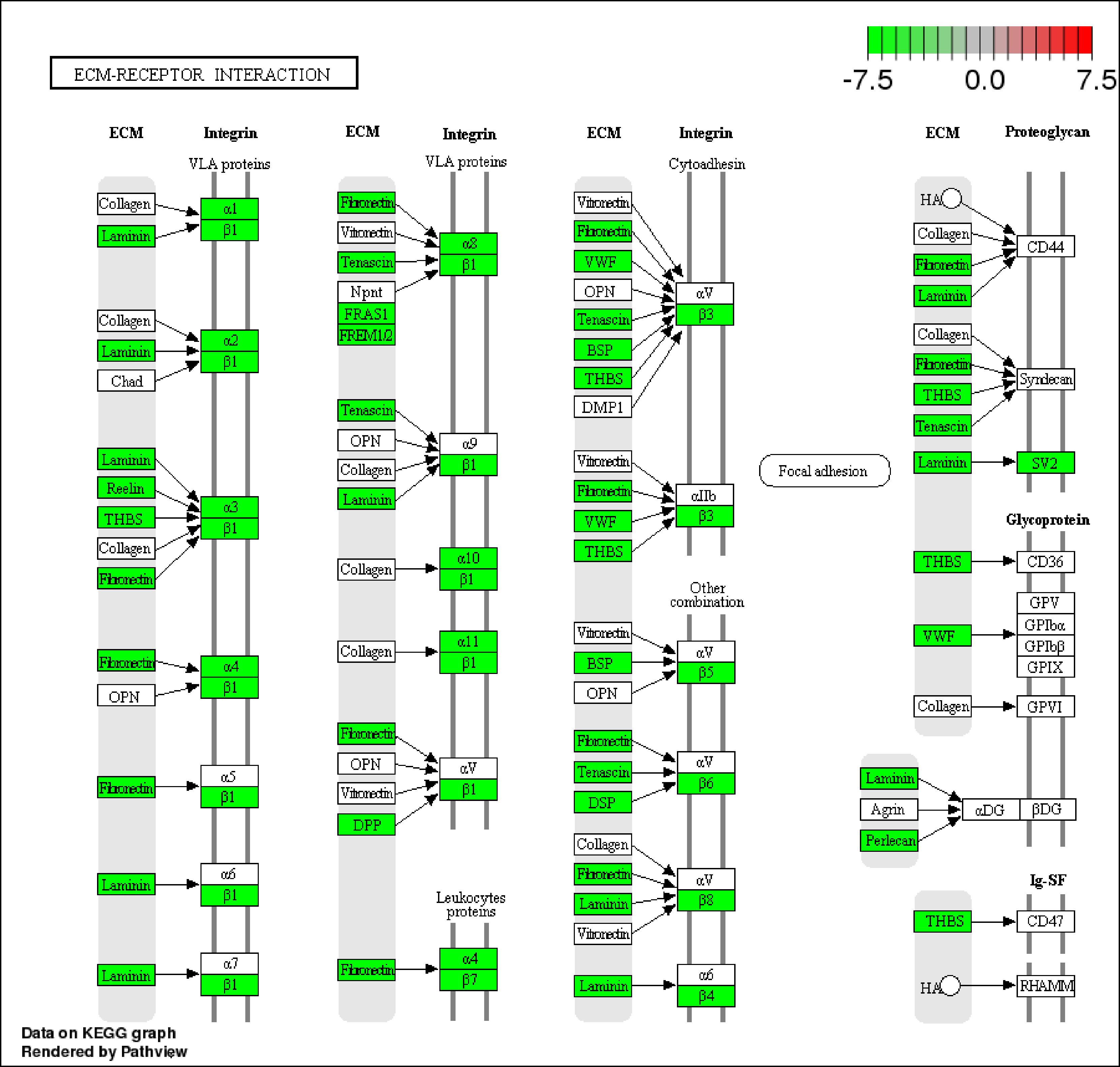
ECM-RECEPTOR INTERACTION
-7.5
0.0
7.5
ECM
Integrin
VLA proteins
Collagen
Laminin
α1
β1
Collagen
Laminin
Chad
α2
β1
Laminin
Reelin
THBS
Collagen
Fibronectin
α3
β1
Fibronectin
OPN
α4
β1
Fibronectin
α5
β1
Laminin
α6
β1
Laminin
α7
β1
ECM
Integrin
VLA proteins
Fibronectin
Vitronectin
Tenascin
Npnt
FRAS1
FREM1/2
α8
β1
Tenascin
OPN
Collagen
Laminin
α9
β1
Collagen
α10
β1
Collagen
α11
β1
Fibronectin
OPN
Vitronectin
DPP
αV
β1
Leukocytes proteins
Fibronectin
α4
β7
ECM
Integrin
Cytoadhesin
Vitronectin
Fibronectin
VWF
OPN
Tenascin
BSP
THBS
DMP1
αV
β3
Vitronectin
Fibronectin
VWF
THBS
αIIb
β3
Other combination
Vitronectin
BSP
OPN
αV
β5
Fibronectin
Tenascin
DSP
αV
β6
Collagen
Fibronectin
Laminin
Vitronectin
αV
β8
Laminin
α6
β4
Focal adhesion
ECM
Proteoglycan
HA
Collagen
Fibronectin
Laminin
CD44
Collagen
Fibronectin
THBS
Tenascin
Syndecan
Laminin
SV2
Glycoprotein
THBS
CD36
VWF
GPV
GPIbα
GPIbβ
GPIX
Collagen
GPVI
Laminin
Agrin
Perlecan
αDG
βDG
Ig-SF
THBS
CD47
HA
RHAMM
Data on KEGG graph
Rendered by Pathview

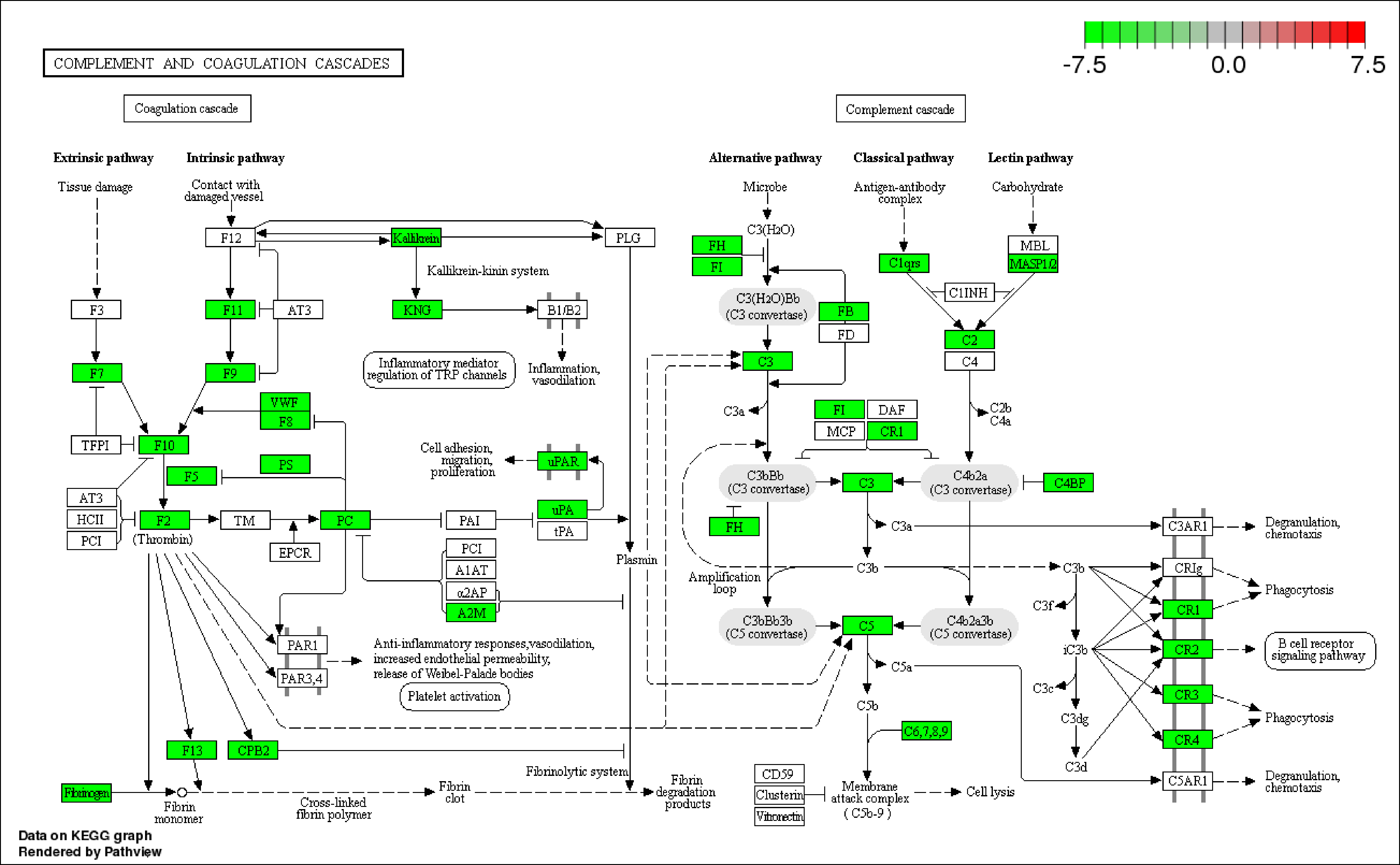

COMPLEMENT AND COAGULATION CASCADES
-7.5
0.0
7.5
Coagulation cascade
Complement cascade
Extrinsic pathway
Intrinsic pathway
Alternative pathway
Classical pathway
Lectin pathway
Tissue damage
Contact with damaged vessel
Microbe
Antigen-antibody complex
Carbohydrate
F12
Kallikrein
PLG
FH
FI
C3(H2O)
MBL
MASP1/2
C1qrs
C1INH
Kallikrein-kinin system
F3
F11
AT3
KNG
B1/B2
C3(H2O)Bb (C3 convertase)
FB
FD
C2
C4
F7
F9
Inflammatory mediator regulation of TRP channels
Inflammation, vasodilation
C3
VWF
F8
C3a
FI
DAF
MCP
CR1
C2b C4a
TFPI
F10
Cell adhesion, migration, proliferation
uPAR
PS
F5
C3bBb (C3 convertase)
C3
C4b2a (C3 convertase)
C4BP
AT3
HCII
PCI
F2
(Thrombin)
TM
PC
PAI
uPA
tPA
EPCR
FH
C3a
C3AR1
Degranulation, chemotaxis
Plasmin
PCI
A1AT
α2AP
A2M
Amplification loop
C3b
C3b
CRIg
Phagocytosis
C3f
CR1
C3bBb3b (C5 convertase)
C5
C4b2a3b (C5 convertase)
iC3b
CR2
B cell receptor signaling pathway
PAR1
PAR3,4
Anti-inflammatory responses,vasodilation, increased endothelial permeability, release of Weibel-Palade bodies
Platelet activation
C5a
C3c
CR3
Phagocytosis
C5b
C3dg
C6,7,8,9
CR4
F13
CPB2
Fibrinolytic system
C3d
CD59
Clusterin
Vitronectin
Membrane attack complex (C5b-9)
Cell lysis
C5AR1
Degranulation, chemotaxis
Fibrinogen
Fibrin monomer
Cross-linked fibrin polymer
Fibrin clot
Fibrin degradation products
Data on KEGG graph
Rendered by Pathview

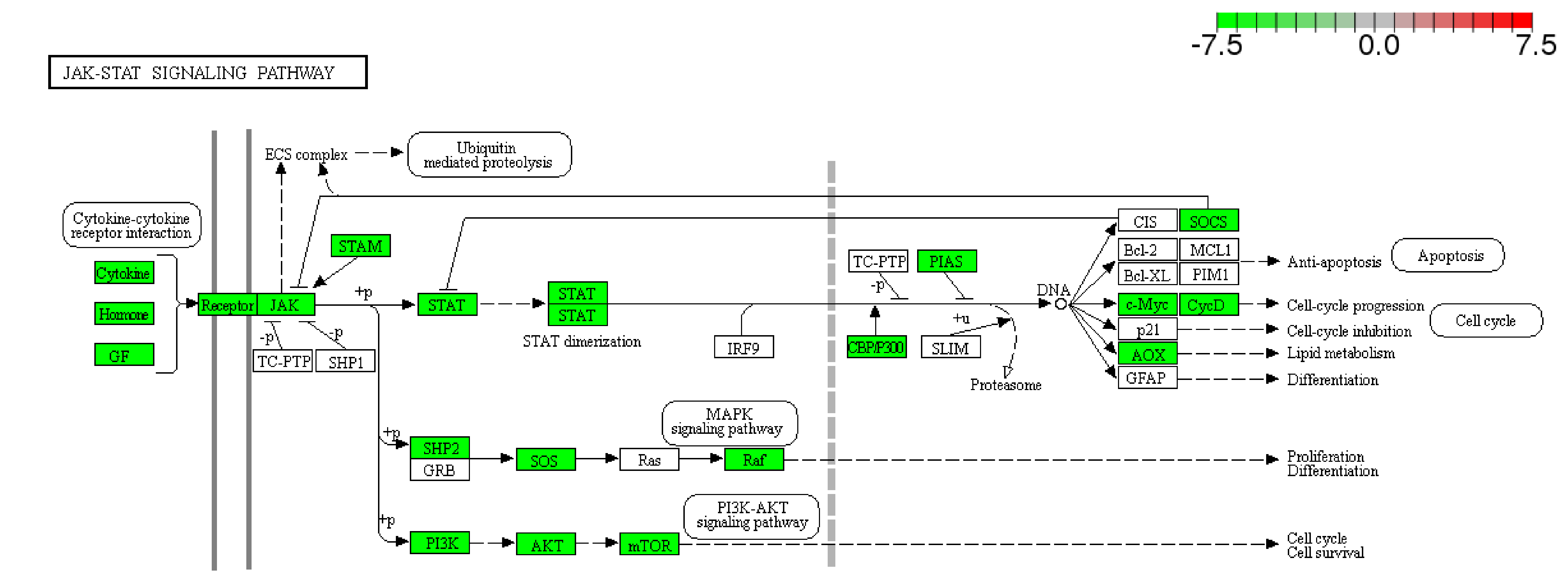

Data on KEGG graph
Rendered by Pathview

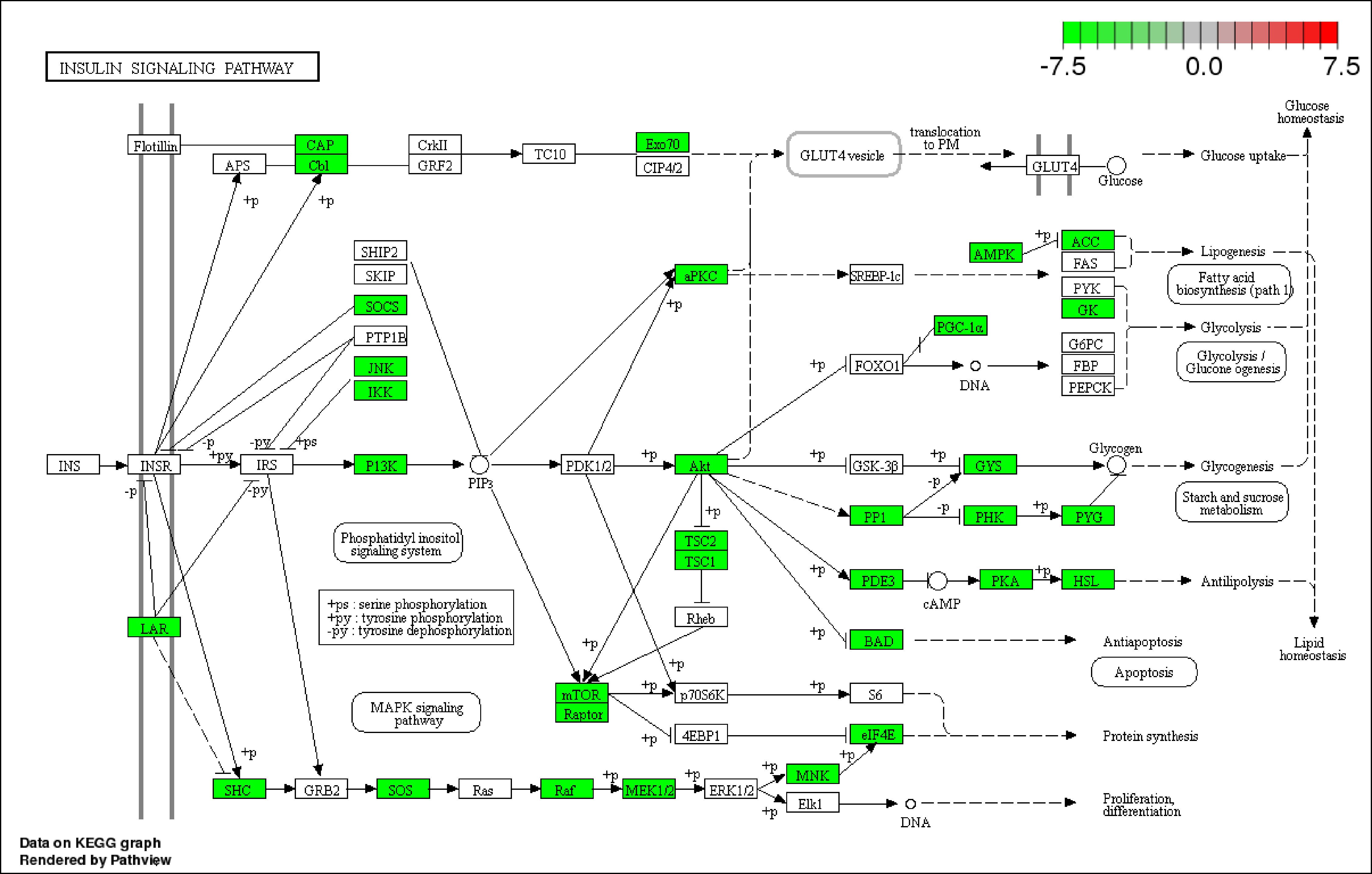

INSULIN SIGNALING PATHWAY
-7.5
0.0
7.5
Flotillin
CAP
Cbl
APS
CrkII
GRF2
TC10
Exo70
CIP4/2
GLUT4 vesicle
translocation to PM
GLUT4
Glucose
Glucose uptake
Glucose homeostasis
SHIP2
SKIP
SOCS
PTP1B
JNK
IKK
aPKC
SREBP-1c
AMPK
ACC
FAS
Lipogenesis
Fatty acid biosynthesis (path 1)
PYK
GK
Glycolysis
Glycolysis / Glucone ogenesis
PGC-1α
FOXO1
DNA
G6PC
FBP
PEPCK
INS
INSR
IRS
PI3K
PIP3
PDK1/2
Akt
GSK-3β
GYS
Glycogen
Glycogenesis
Starch and sucrose metabolism
PP1
PHK
PYG
TSC2
TSC1
Rheb
PDE3
cAMP
PKA
HSL
Antilipolysis
Lipid homeostasis
BAD
Antiapoptosis
Apoptosis
Phosphatidyl inositol signaling system
+ps : serine phosphorylation
+py : tyrosine phosphorylation
-py : tyrosine dephosphorylation
LAR
mTOR
Raptor
p70S6K
S6
4EBP1
eIF4E
Protein synthesis
MAPK signaling pathway
SHC
GRB2
SOS
Ras
Raf
MEK1/2
ERK1/2
MNK
Elk1
DNA
Proliferation, differentiation
+p
-p
+py
-py
+ps
Data on KEGG graph
Rendered by Pathview
