# Supplementary material for: In silico predictions of protein interactions between Zika virus and human host
Source: PeerJ. 2021 Aug 24;9:e11770. doi: 10.7717/peerj.11770 (PMC8395582; doi:10.7717/peerj.11770)
Supplement: Supplemental Information 5 [file peerj-09-11770-s005.pdf]

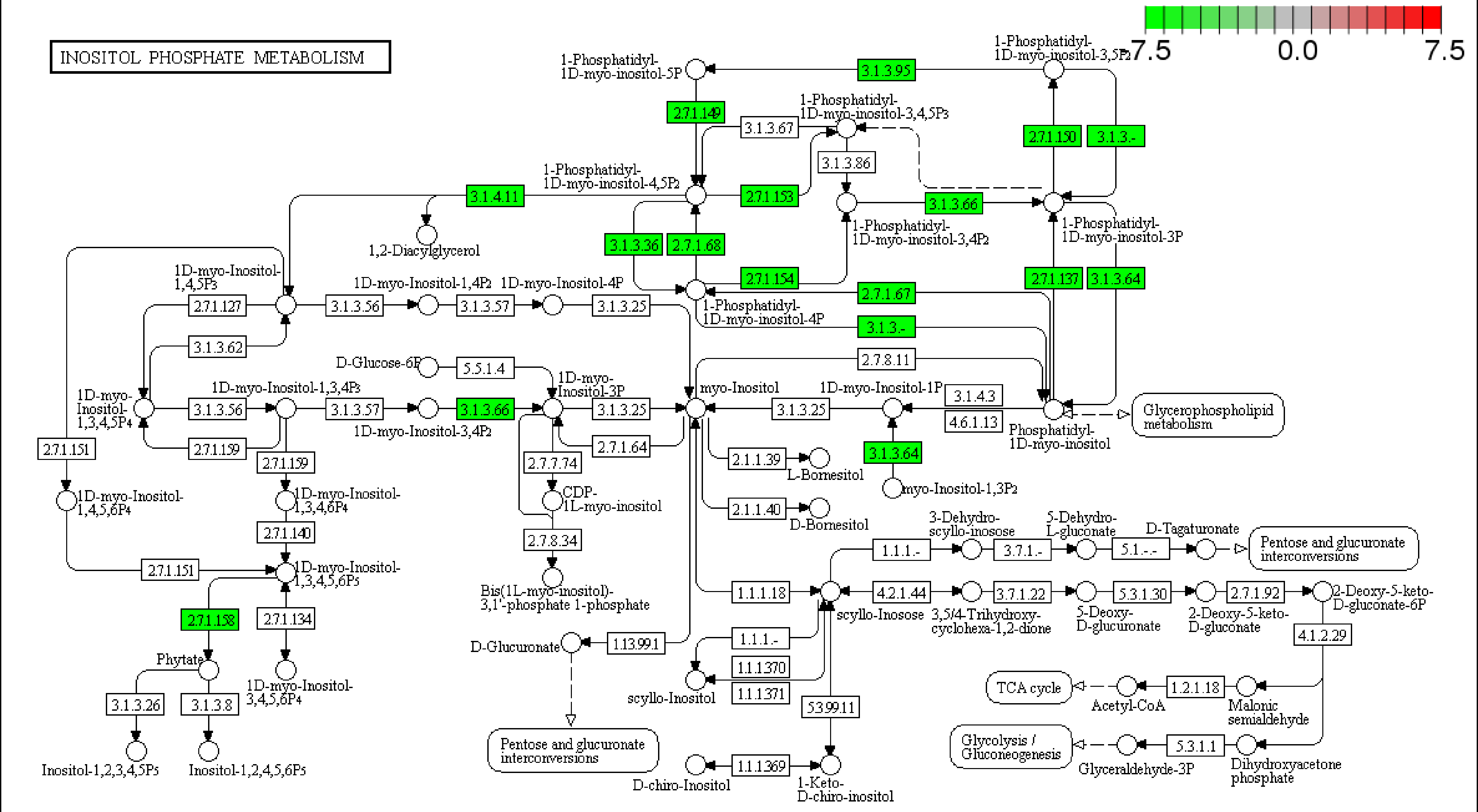

Data on KEGG graph
Rendered by Pathview

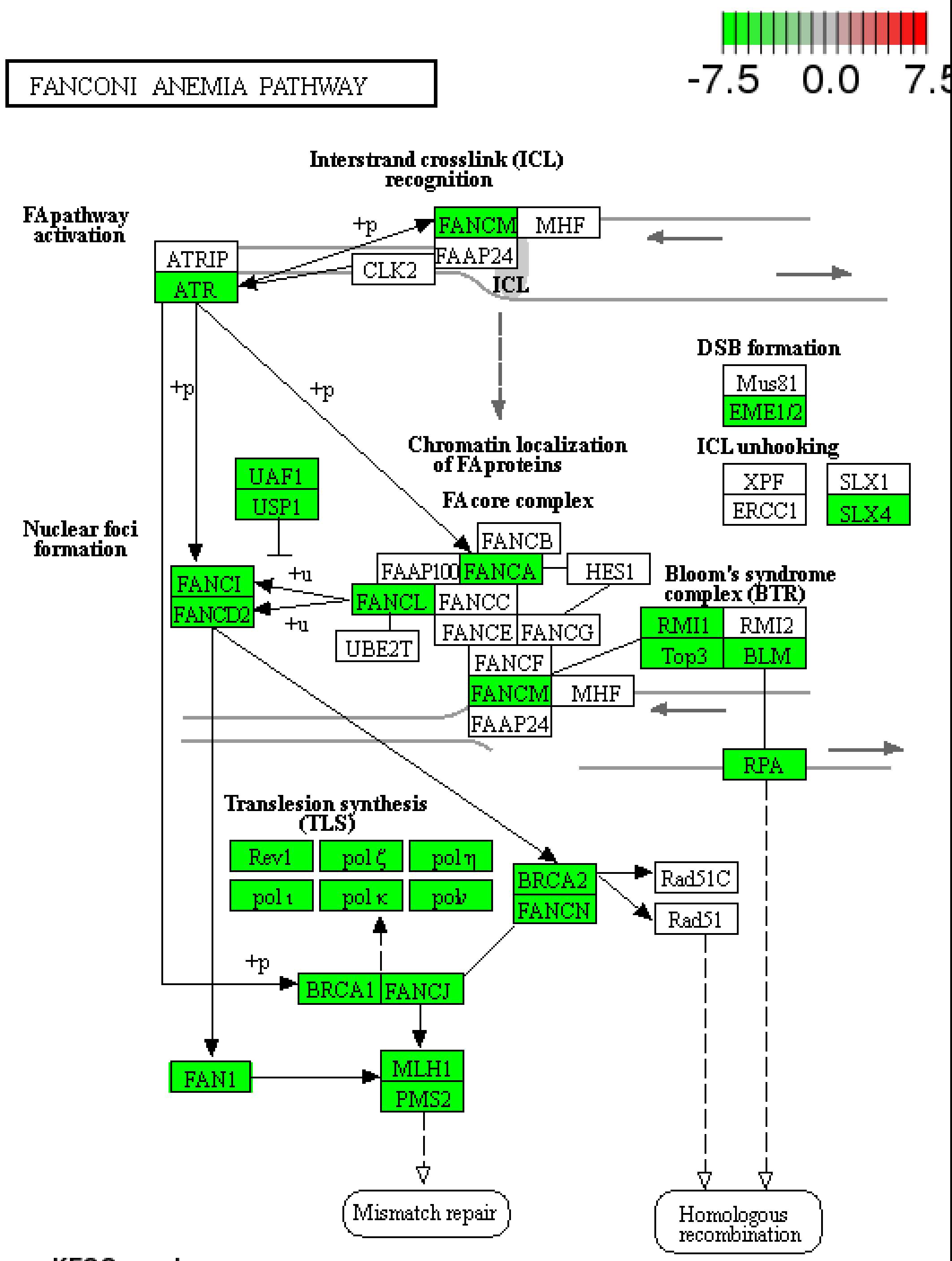

Data on KEGG graph
Rendered by Pathview

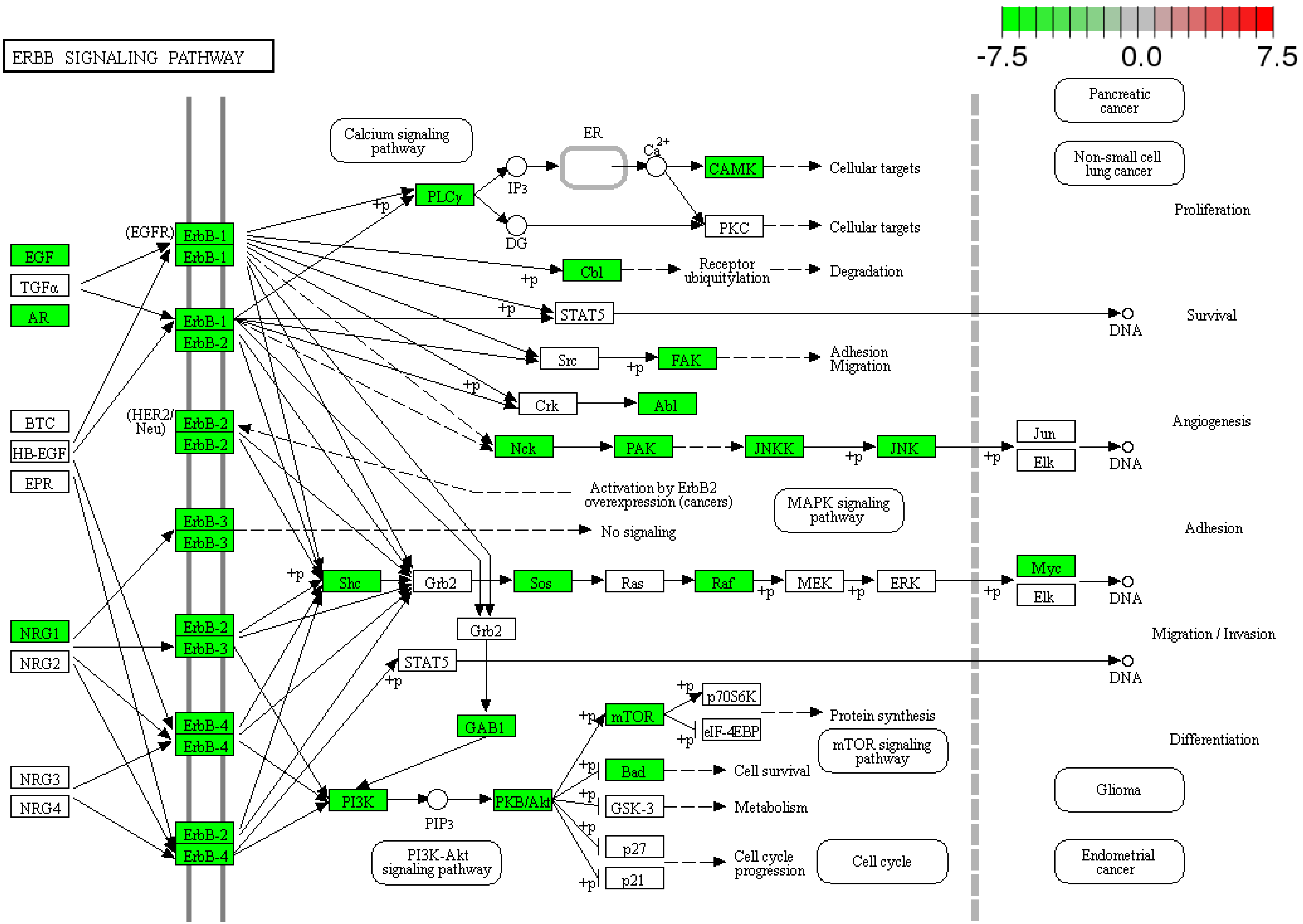

Data on KEGG graph
Rendered by Pathview

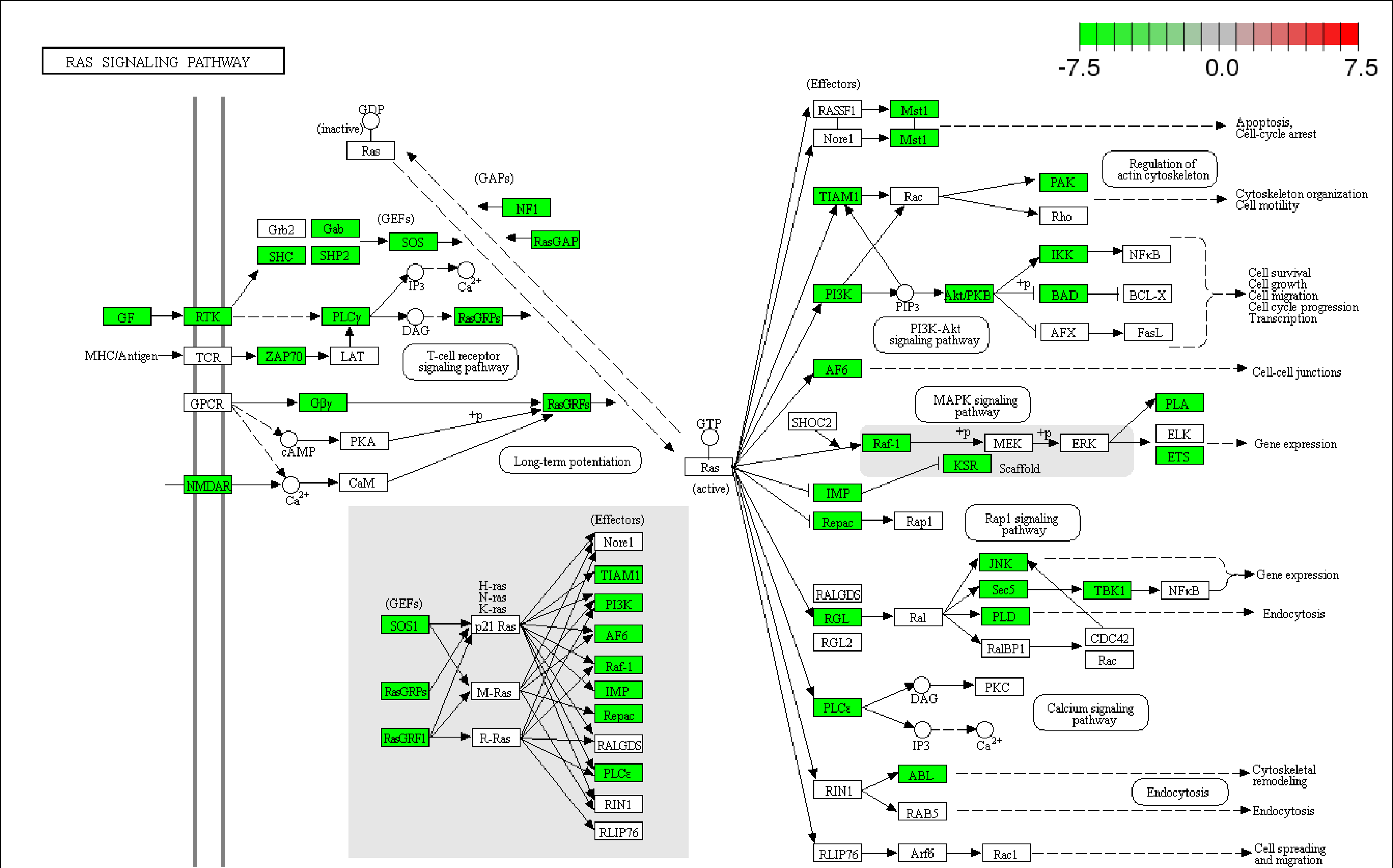

Data on KEGG graph
Rendered by Pathview

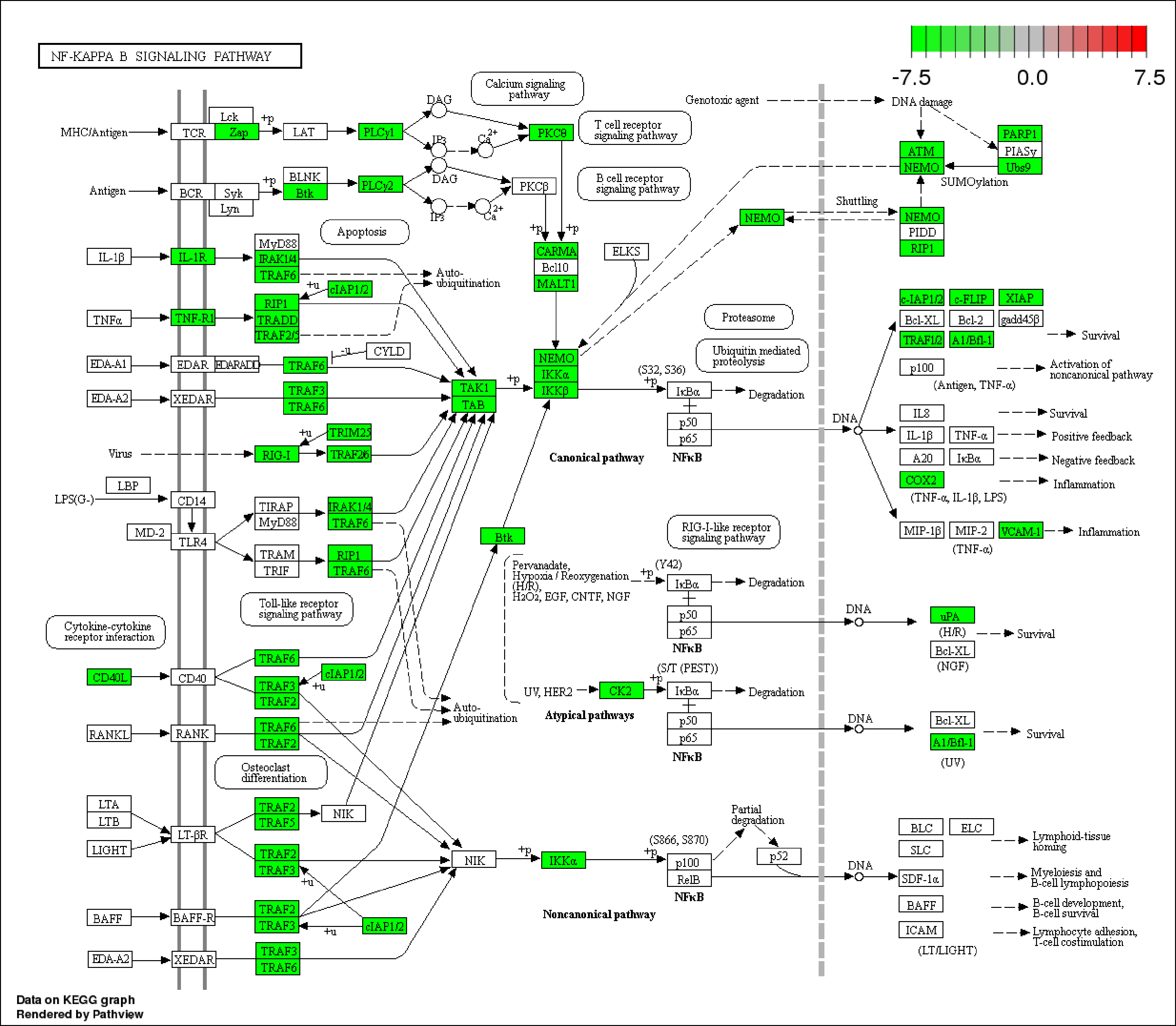

NF-KAPPA B SIGNALING PATHWAY
-7.5
0.0
7.5
Calcium signaling pathway
T cell receptor signaling pathway
B cell receptor signaling pathway
Genotoxic agent
DNA damage
MHC/Antigen
TCR
Lck
Zap
LAT
PLCγ1
DAG
IP3
PKCθ
Antigen
BCR
Syk
Lyn
BLNK
Btk
PLCγ2
PKCβ
PARP1
PIASy
Ubs9
ATM
NEMO
SUMOylation
Shuttling
PIDD
RIP1
IL-1β
IL-1R
MyD88
IRAK1/4
TRAF6
Apoptosis
Auto-ubiquitination
cIAP1/2
TNFα
TNF-R1
RIP1
TRADD
TRAF2/5
CARMA
Bcl10
MALT1
ELKS
Proteasome
Ubiquitin mediated proteolysis
EDA-A1
EDAR
EDARADD
CYLD
EDA-A2
XEDAR
TRAF3
TAK1
TAB
IKKα
IKKβ
(S32, S36)
IκBα
Degradation
p50
p65
NFκB
Canonical pathway
Virus
TRIM25
RIG-I
TRAF2/6
LBP
LPS(G-)
CD14
MD-2
TLR4
TIRAP
TRAM
TRIF
Btk
RIG-I-like receptor signaling pathway
Pervanadate, Hypoxia / Reoxygenation (H/R), H2O2, EGF, CNTF, NGF
(Y42)
Toll-like receptor signaling pathway
Cytokine-cytokine receptor interaction
CD40L
CD40
TRAF2
UV, HER2
CK2
(S/T (PEST))
Atypical pathways
RANKL
RANK
Osteoclast differentiation
LTA
LTB
LIGHT
LT-βR
NIK
BAFF
BAFF-R
Partial degradation
(S866, S870)
p100
RelB
p52
Noncanonical pathway
DNA
c-IAP1/2
c-FLIP
XIAP
Bcl-XL
Bcl-2
gadd45β
TRAF1/2
A1/Bfl-1
Survival
p100
Activation of noncanonical pathway
(Antigen, TNF-α)
IL8
IL-1β
TNF-α
Positive feedback
A20
Negative feedback
COX2
Inflammation
(TNF-α, IL-1β, LPS)
MIP-1β
MIP-2
VCAM-1
(TNF-α)
uPA
(H/R)
(NGF)
(UV)
BLC
ELC
SLC
SDF-1α
ICAM
Lymphoid-tissue homing
Myeloiesis and B-cell lymphopoiesis
B-cell development, B-cell survival
Lymphocyte adhesion, T-cell costimulation
(LT/LIGHT)
Data on KEGG graph
Rendered by Pathview

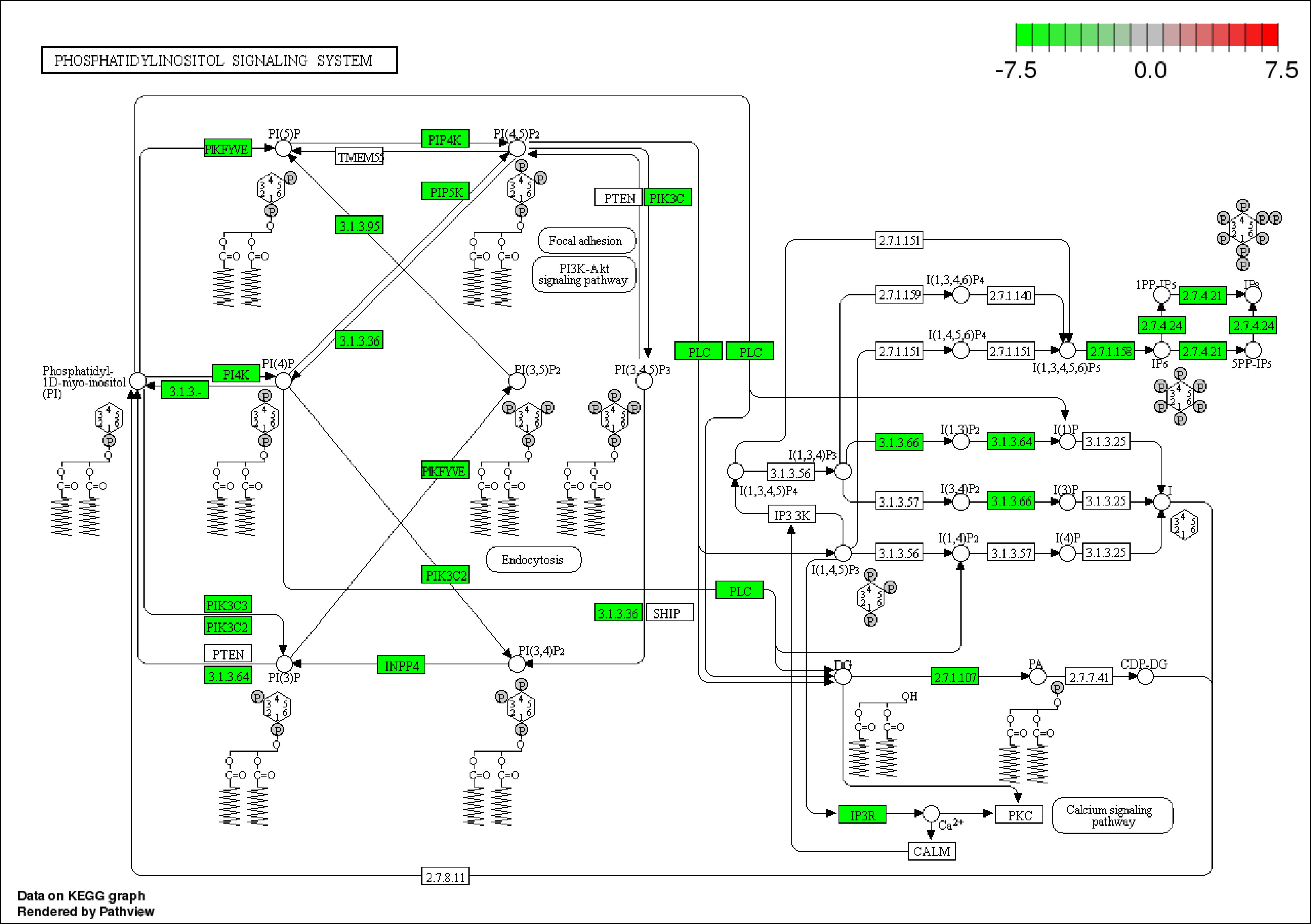
PHOSPHATIDYLINOSITOL SIGNALING SYSTEM
-7.5
0.0
7.5
PI(5)P
PI(4,5)P2
PIKFYVE
PIP4K
TMEM55
PIP5K
PTEN
PIK3C
Focal adhesion
PI3K-Akt signaling pathway
3.1.3.95
3.1.3.36
Phosphatidyl-1D-myo-inositol (PI)
PI4K
3.1.3.-
PI(4)P
PI(3,5)P2
PI(3,4,5)P3
PLC
PIKFYVE
Endocytosis
PIK3C2
PIK3C3
PIK3C2
PTEN
3.1.3.64
PI(3)P
INPP4
PI(3,4)P2
3.1.3.36
SHIP
2.7.1.151
2.7.1.159
I(1,3,4,6)P4
2.7.1.140
I(1,4,5,6)P4
2.7.1.151
I(1,3,4,5,6)P5
2.7.1.158
1PP-IP5
2.7.4.21
IP8
2.7.4.24
2.7.4.24
IP6
2.7.4.21
5PP-IP5
I(1,3,4)P3
3.1.3.56
I(1,3,4,5)P4
IP3 3K
3.1.3.66
I(1,3)P2
3.1.3.64
I(1)P
3.1.3.25
I(3,4)P2
3.1.3.57
3.1.3.66
I(3)P
3.1.3.25
I
I(1,4,5)P3
3.1.3.56
I(1,4)P2
3.1.3.57
I(4)P
3.1.3.25
DG
2.7.1.107
PA
2.7.7.41
CDP-DG
IP3R
Ca2+
PKC
Calcium signaling pathway
CALM
2.7.8.11
Data on KEGG graph
Rendered by Pathview

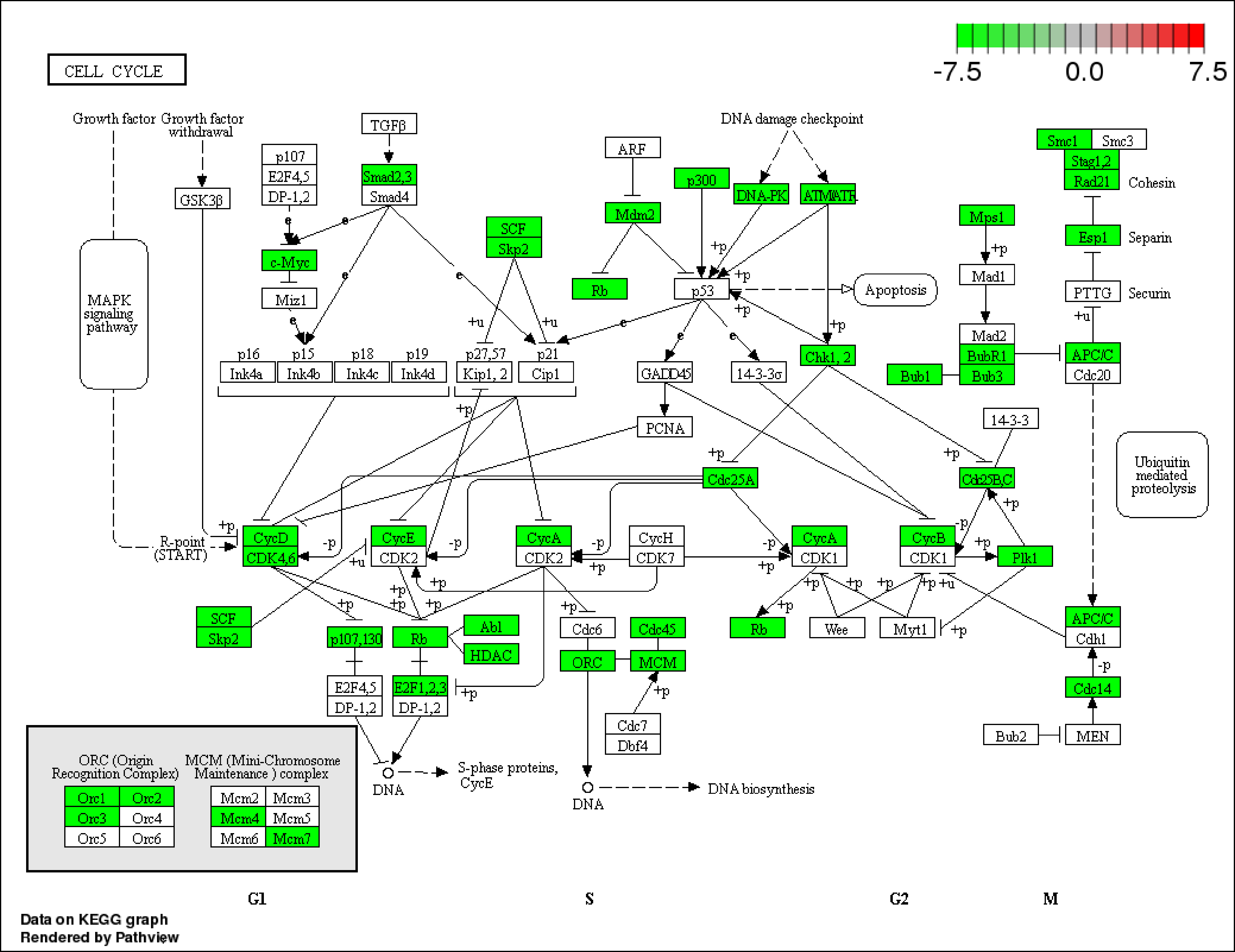
CELL CYCLE
-7.5
0.0
7.5
Growth factor
Growth factor withdrawal
MAPK signaling pathway
GSK3β
R-point (START)
p107
E2F4,5
DP-1,2
TGFβ
Smad2,3
Smad4
c-Myc
Miz1
SCF
Skp2
ARF
Mdm2
Rb
p300
DNA-PK
ATM/ATR
DNA damage checkpoint
p53
Apoptosis
GADD45
14-3-3σ
PCNA
Chk1, 2
p16
Ink4a
p15
Ink4b
p18
Ink4c
p19
Ink4d
p27,57
Kip1, 2
p21
Cip1
CycD
CDK4,6
CycE
CDK2
CycA
CDK2
CycH
CDK7
CycA
CDK1
CycB
CDK1
Cdc25A
Cdc25B,C
14-3-3
Plk1
Mps1
Mad1
Mad2
BubR1
Bub3
Bub1
APC/C
Cdc20
Smc1
Smc3
Stag1,2
Rad21
Cohesin
Esp1
Separin
PTTG
Securin
Ubiquitin mediated proteolysis
SCF
Skp2
p107,130
E2F4,5
DP-1,2
Rb
Abl
HDAC
E2F1,2,3
DP-1,2
DNA
S-phase proteins, CycE
Cdc6
Cdc45
ORC
MCM
Cdc7
Dbf4
DNA
DNA biosynthesis
Rb
Wee
Myt1
APC/C
Cdh1
Cdc14
Bub2
MEN
ORC (Origin Recognition Complex)
Orc1
Orc2
Orc3
Orc4
Orc5
Orc6
MCM (Mini-Chromosome Maintenance) complex
Mcm2
Mcm3
Mcm4
Mcm5
Mcm6
Mcm7
G1
S
G2
M
Data on KEGG graph
Rendered by Pathview

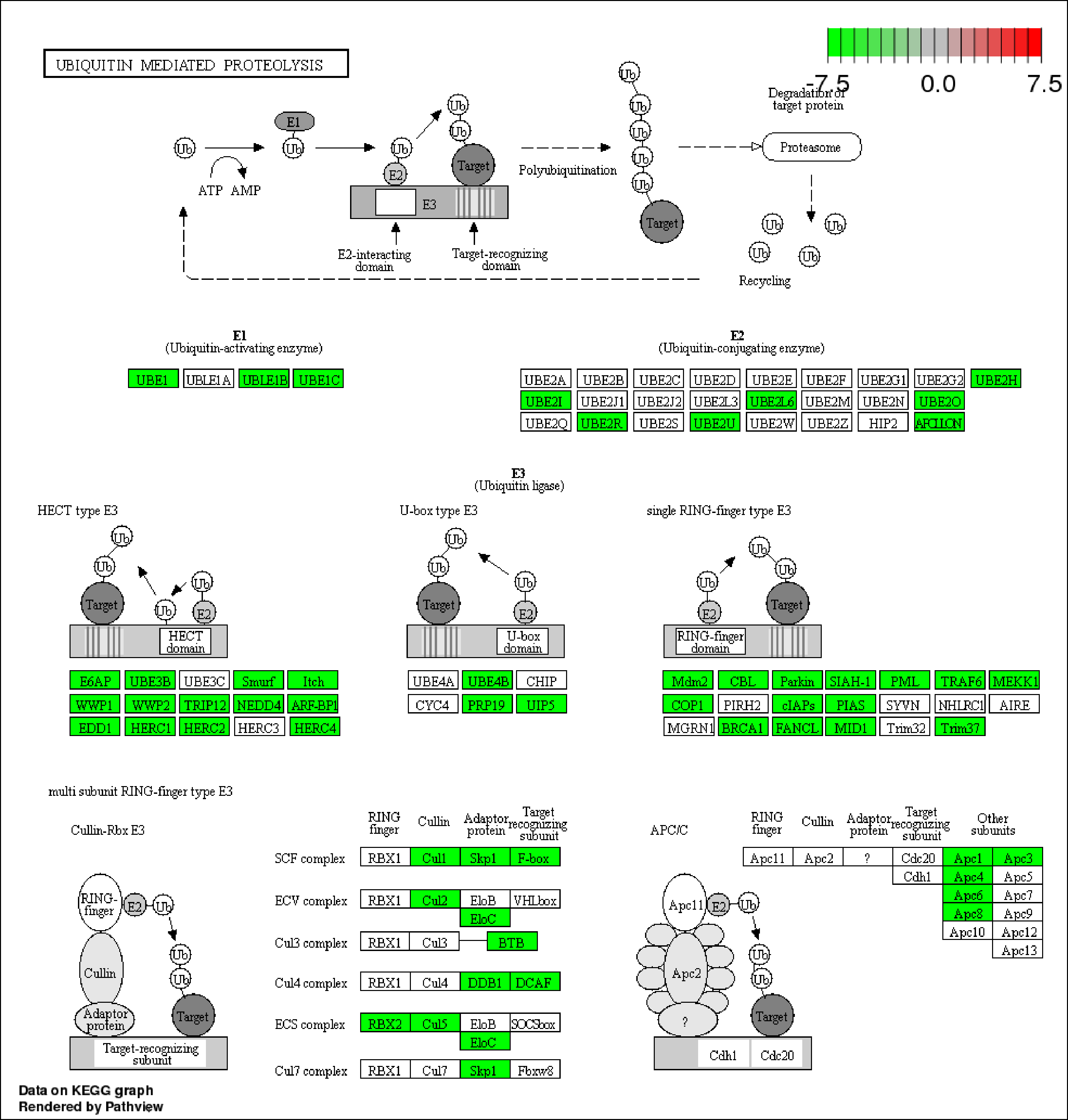
UBIQUITIN MEDIATED PROTEOLYSIS
-7.5
0.0
7.5
Ub
E1
ATP AMP
E2
Target
E3
E2-interacting domain
Target-recognizing domain
Polyubiquitination
Degradation of target protein
Proteasome
Recycling
E1
(Ubiquitin-activating enzyme)
UBE1
UBLE1A
UBLE1B
UBLE1C
E2
(Ubiquitin-conjugating enzyme)
UBE2A
UBE2B
UBE2C
UBE2D
UBE2E
UBE2F
UBE2G1
UBE2G2
UBE2H
UBE2I
UBE2J1
UBE2J2
UBE2L3
UBE2L6
UBE2M
UBE2N
UBE2O
UBE2Q
UBE2R
UBE2S
UBE2U
UBE2W
UBE2Z
HIP2
APOLLON
E3
(Ubiquitin ligase)
HECT type E3
HECT domain
E6AP
UBE3B
UBE3C
Smurf
Itch
WWP1
WWP2
TRIP12
NEDD4
ARF-BP1
EDD1
HERC1
HERC2
HERC3
HERC4
U-box type E3
U-box domain
UBE4A
UBE4B
CHIP
CYC4
PRP19
UIP5
single RING-finger type E3
RING-finger domain
Mdm2
CBL
Parkin
SIAH-1
PML
TRAF6
MEKK1
COP1
PIRH2
cIAPs
PIAS
SYVN
NHLRC1
AIRE
MGRN1
BRCA1
FANCL
MID1
Trim32
Trim37
multi subunit RING-finger type E3
Cullin-Rbx E3
RING-finger
Cullin
Adaptor protein
Target-recognizing subunit
RING finger
Cullin
Adaptor protein
Target recognizing subunit
SCF complex
RBX1
Cul1
Skp1
F-box
ECV complex
RBX1
Cul2
EloB
VHLbox
EloC
Cul3 complex
RBX1
Cul3
BTB
Cul4 complex
RBX1
Cul4
DDB1
DCAF
ECS complex
RBX2
Cul5
EloB
SOCSbox
EloC
Cul7 complex
RBX1
Cul7
Skp1
Fbxw8
APC/C
RING finger
Cullin
Adaptor protein
Target recognizing subunit
Other subunits
Apc11
Apc2
?
Cdc20
Cdh1
Apc1
Apc3
Apc4
Apc5
Apc6
Apc7
Apc8
Apc9
Apc10
Apc12
Apc13
Cdh1
Cdc20
Data on KEGG graph
Rendered by Pathview

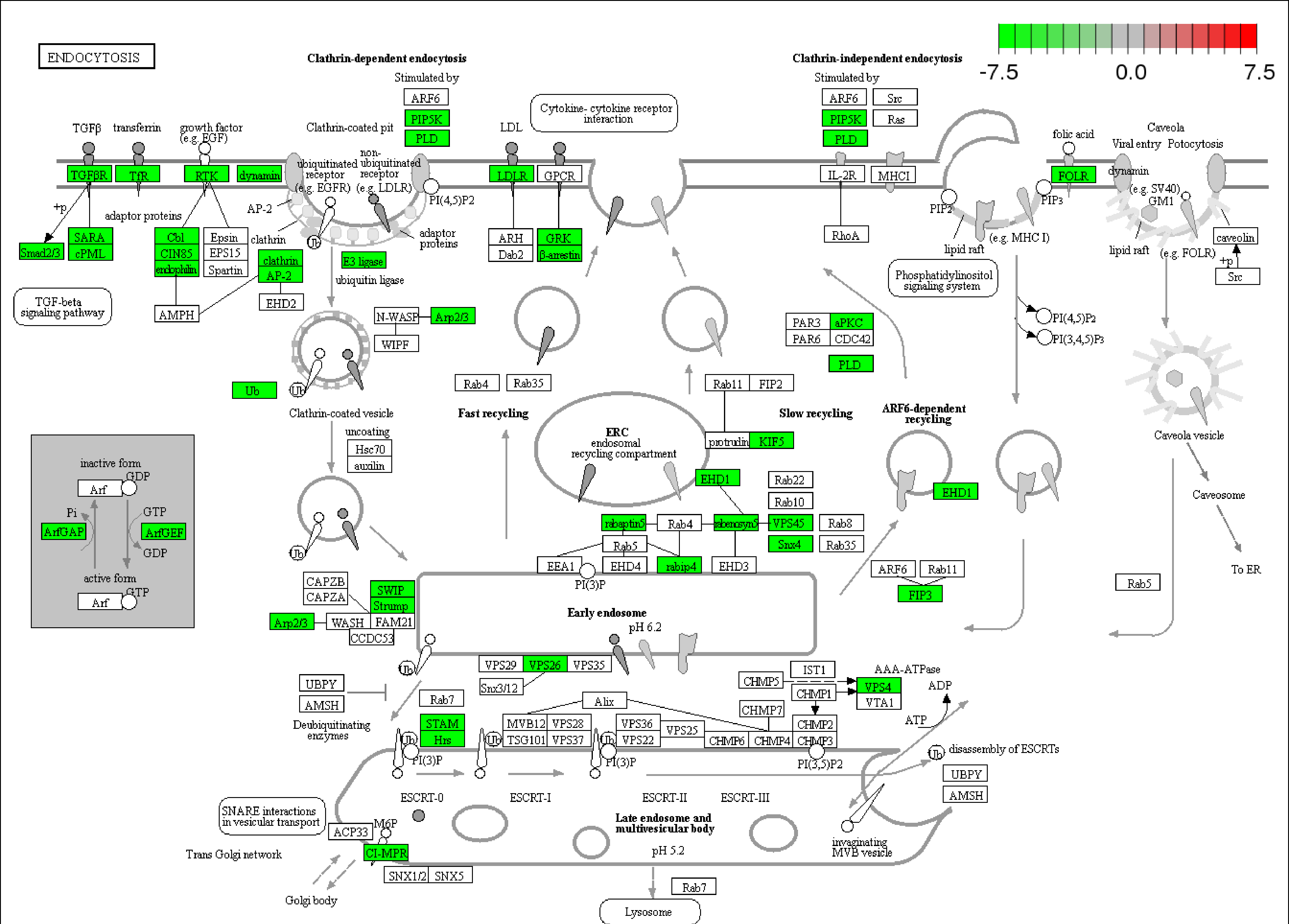

Data on KEGG graph
Rendered by Pathview

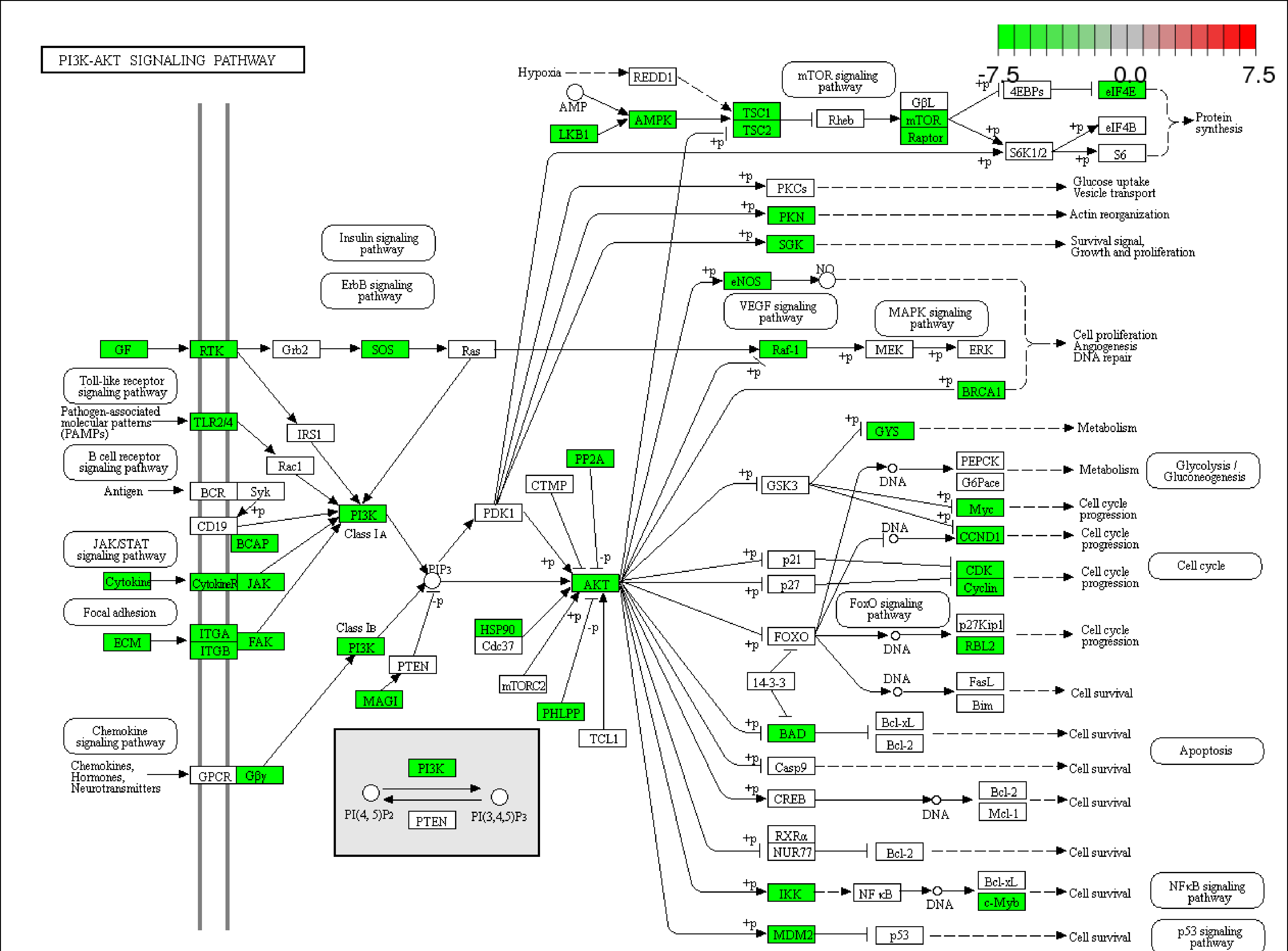

Data on KEGG graph
Rendered by Pathview

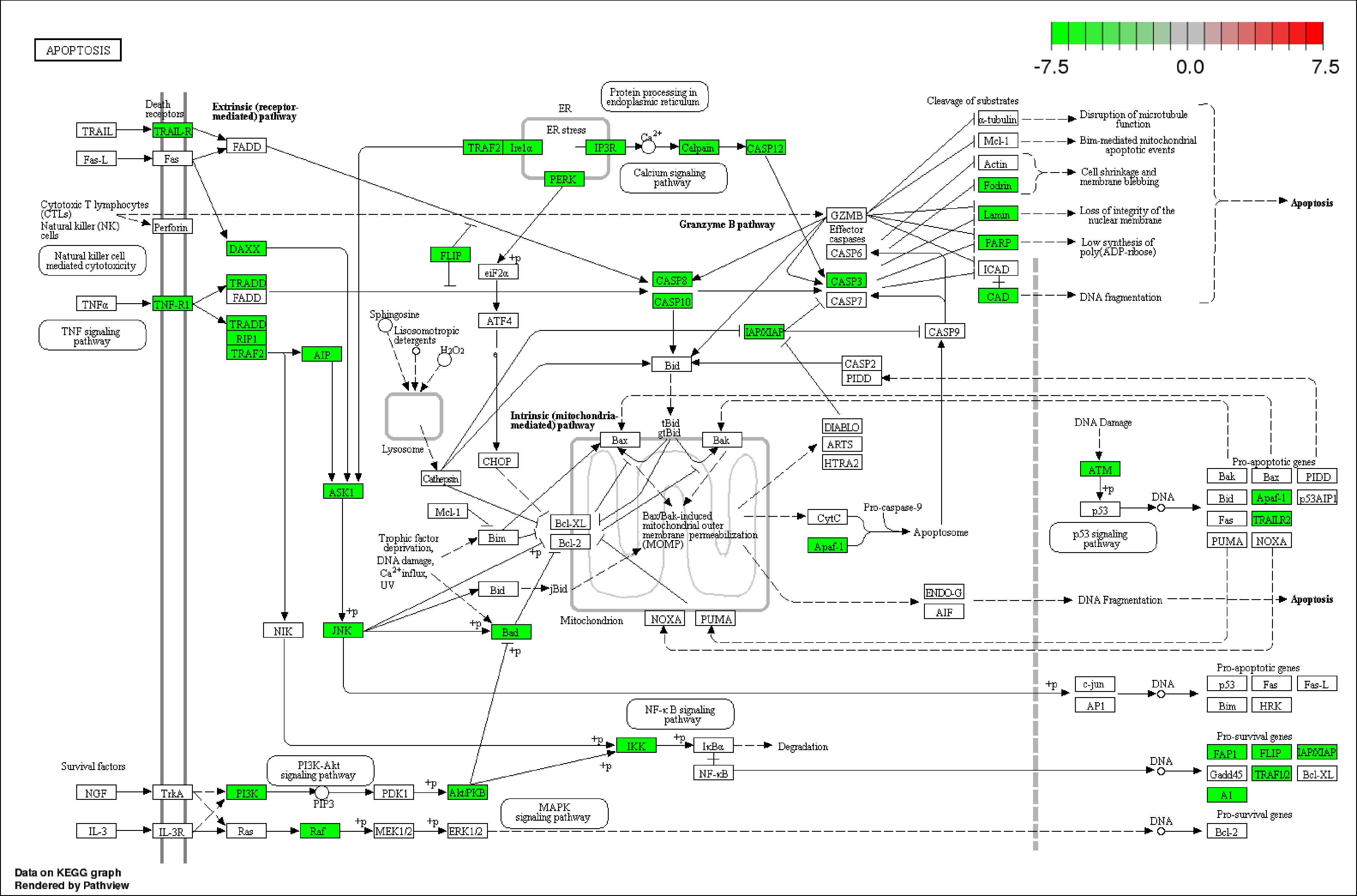

APOPTOSIS
-7.5
0.0
7.5
Death receptors
Extrinsic (receptor-mediated) pathway
TRAIL
TRAIL-R
Fas-L
Fas
FADD
Cytotoxic T lymphocytes (CTLs)
Natural killer (NK) cells
Perforin
Natural killer cell mediated cytotoxicity
DAXX
TNFα
TNF-R1
TRADD
FADD
TRADD
RIP1
TRAF2
TNF signaling pathway
AIP
ASK1
JNK
NIK
ER
ER stress
TRAF2
Ire1α
IP3R
Ca2+
Calpain
CASP12
PERK
Protein processing in endoplasmic reticulum
Calcium signaling pathway
FLIP
eIF2α
ATF4
CHOP
Sphingosine
Lisosomotropic detergents
H2O2
Lysosome
Cathepsin
Mcl-1
Bim
Trophic factor deprivation, DNA damage, Ca2+ influx, UV
Bid
jBid
Bad
Granzyme B pathway
GZMB
Effector caspases
CASP6
CASP3
CASP7
CASP8
CASP10
IAP/XIAP
CASP9
Bid
CASP2
PIDD
Intrinsic (mitochondria-mediated) pathway
tBid
gtBid
Bax
Bak
Bcl-XL
Bcl-2
Bax/Bak-induced mitochondrial outer membrane permeabilization (MOMP)
Mitochondrion
NOXA
PUMA
DIABLO
ARTS
HTRA2
CytC
Apaf-1
Pro-caspase-9
Apoptosome
ENDO-G
AIF
DNA Fragmentation
Cleavage of substrates
α-tubulin
Mcl-1
Actin
Fodrin
Lamin
PARP
ICAD
CAD
Disruption of microtubule function
Bim-mediated mitochondrial apoptotic events
Cell shrinkage and membrane blebbing
Loss of integrity of the nuclear membrane
Low synthesis of poly(ADP-ribose)
DNA fragmentation
Apoptosis
DNA Damage
ATM
p53
DNA
p53 signaling pathway
Pro-apoptotic genes
Bak
Bax
PIDD
Bid
Apaf-1
p53AIP1
Fas
TRAILR2
PUMA
NOXA
Apoptosis
c-jun
AP1
Pro-apoptotic genes
p53
Fas
Fas-L
Bim
HRK
NF-κB signaling pathway
IKK
IκBα
NF-κB
Degradation
Pro-survival genes
FAP1
FLIP
IAP/XIAP
Gadd45
TRAF1/2
Bcl-XL
A1
Pro-survival genes
Bcl-2
Survival factors
NGF
TrkA
PI3K
PIP3
PDK1
Akt/PKB
PI3K-Akt signaling pathway
IL-3
IL-3R
Ras
Raf
MEK1/2
ERK1/2
MAPK signaling pathway
+p
Data on KEGG graph
Rendered by Pathview

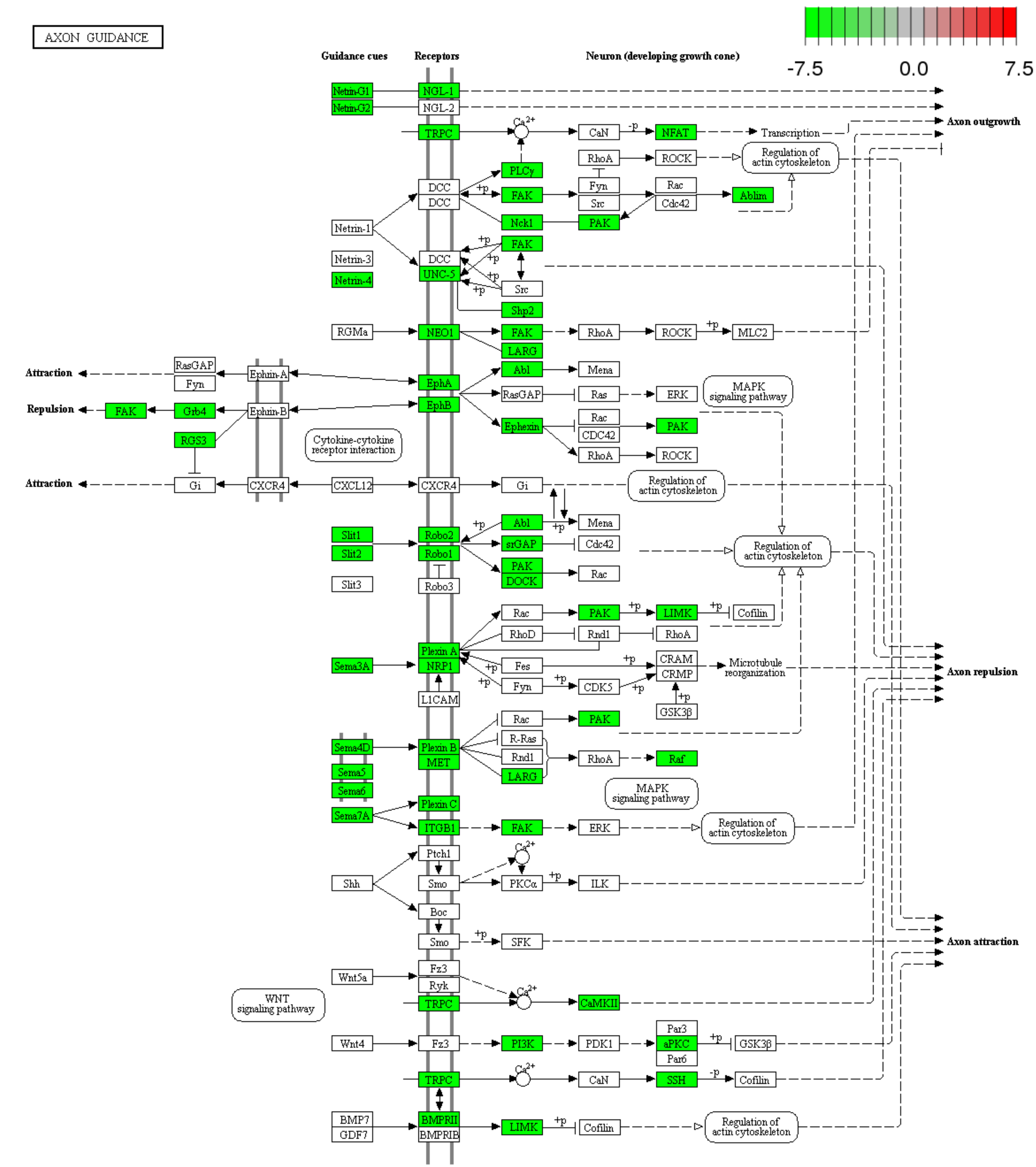

Data on KEGG graph
Rendered by Pathview

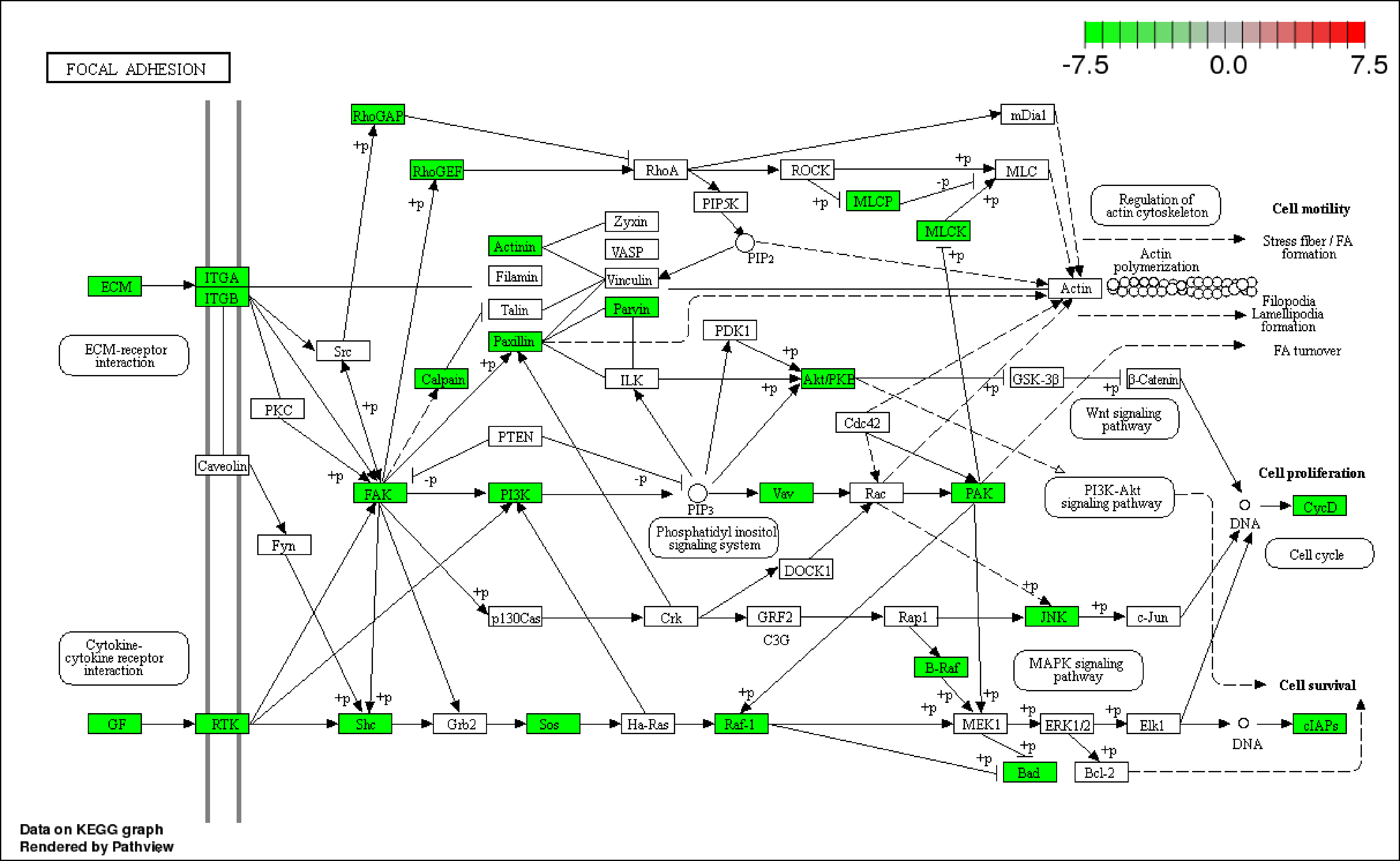

-7.5
0.0
7.5
FOCAL ADHESION
RhoGAP
RhoGEF
RhoA
mDia1
ROCK
MLC
PIP5K
MLCP
MLCK
Regulation of actin cytoskeleton
Cell motility
Stress fiber / FA formation
Zyxin
VASP
Actinin
Filamin
Vinculin
Talin
Parvin
Paxillin
PIP2
Actin
Actin polymerization
Filopodia Lamellipodia formation
FA turnover
ECM
ITGA
ITGB
Src
ECM-receptor interaction
PDK1
Akt/PKB
ILK
GSK-3β
β-Catenin
Calpain
PKC
Wnt signaling pathway
Cdc42
PTEN
Caveolin
FAK
PI3K
PIP3
Vav
Rac
PAK
PI3K-Akt signaling pathway
Cell proliferation
DNA
CycD
Phosphatidyl inositol signaling system
Fyn
DOCK1
Cell cycle
p130Cas
Crk
GRF2
C3G
Rap1
JNK
c-Jun
Cytokine-cytokine receptor interaction
B-Raf
MAPK signaling pathway
Cell survival
GF
RTK
Shc
Grb2
Sos
Ha-Ras
Raf-1
MEK1
ERK1/2
Elk1
cIAPs
Bad
Bcl-2
+p
-p
Data on KEGG graph
Rendered by Pathview

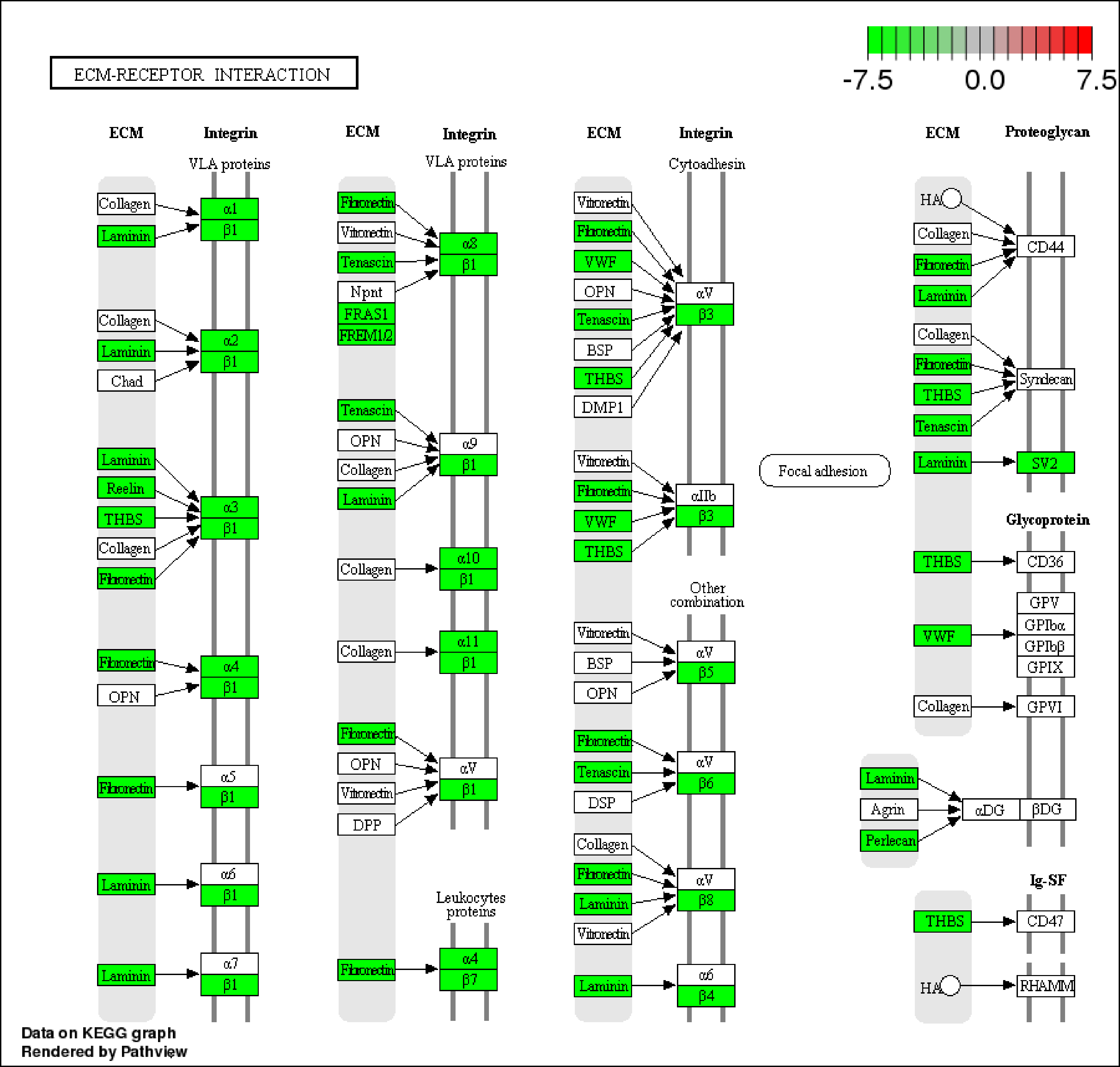
ECM-RECEPTOR INTERACTION
-7.5
0.0
7.5
ECM
Integrin
VLA proteins
Collagen
Laminin
α1
β1
Collagen
Laminin
Chad
α2
β1
Laminin
Reelin
THBS
Collagen
Fibronectin
α3
β1
Fibronectin
OPN
α4
β1
Fibronectin
α5
β1
Laminin
α6
β1
Laminin
α7
β1
ECM
Integrin
VLA proteins
Fibronectin
Vitronectin
Tenascin
Npnt
FRAS1
FREM1/2
α8
β1
Tenascin
OPN
Collagen
Laminin
α9
β1
Collagen
α10
β1
Collagen
α11
β1
Fibronectin
OPN
Vitronectin
DPP
αV
β1
Leukocytes proteins
Fibronectin
α4
β7
ECM
Integrin
Cytoadhesin
Vitronectin
Fibronectin
VWF
OPN
Tenascin
BSP
THBS
DMP1
αV
β3
Vitronectin
Fibronectin
VWF
THBS
αIIb
β3
Other combination
Vitronectin
BSP
OPN
αV
β5
Fibronectin
Tenascin
DSP
αV
β6
Collagen
Fibronectin
Laminin
Vitronectin
αV
β8
Laminin
α6
β4
Focal adhesion
ECM
Proteoglycan
HA
Collagen
Fibronectin
Laminin
CD44
Collagen
Fibronectin
THBS
Tenascin
Syndecan
Laminin
SV2
Glycoprotein
THBS
CD36
VWF
GPV
GPIbα
GPIbβ
GPIX
Collagen
GPVI
Laminin
Agrin
Perlecan
αDG
βDG
Ig-SF
THBS
CD47
HA
RHAMM
Data on KEGG graph
Rendered by Pathview

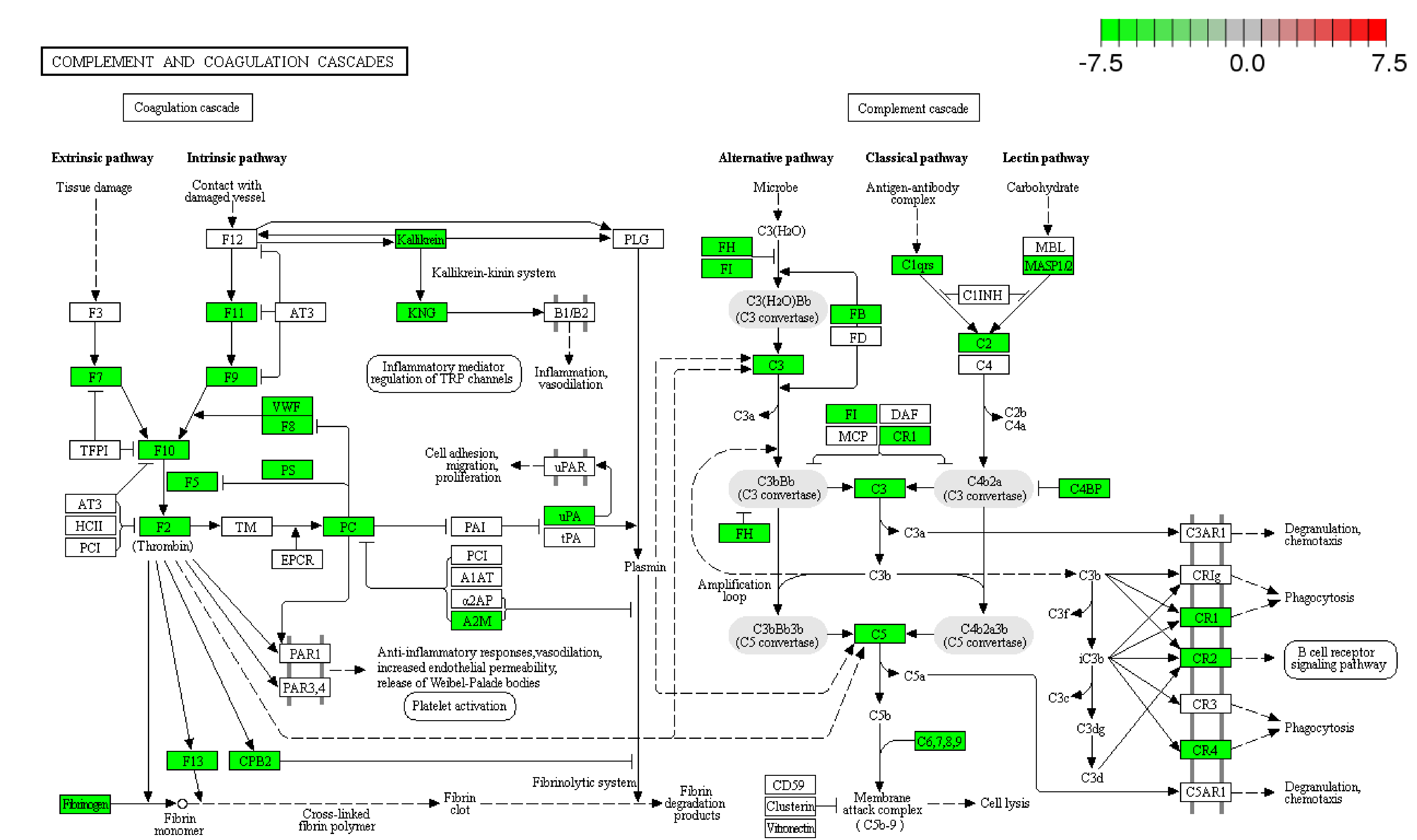

Data on KEGG graph
Rendered by Pathview

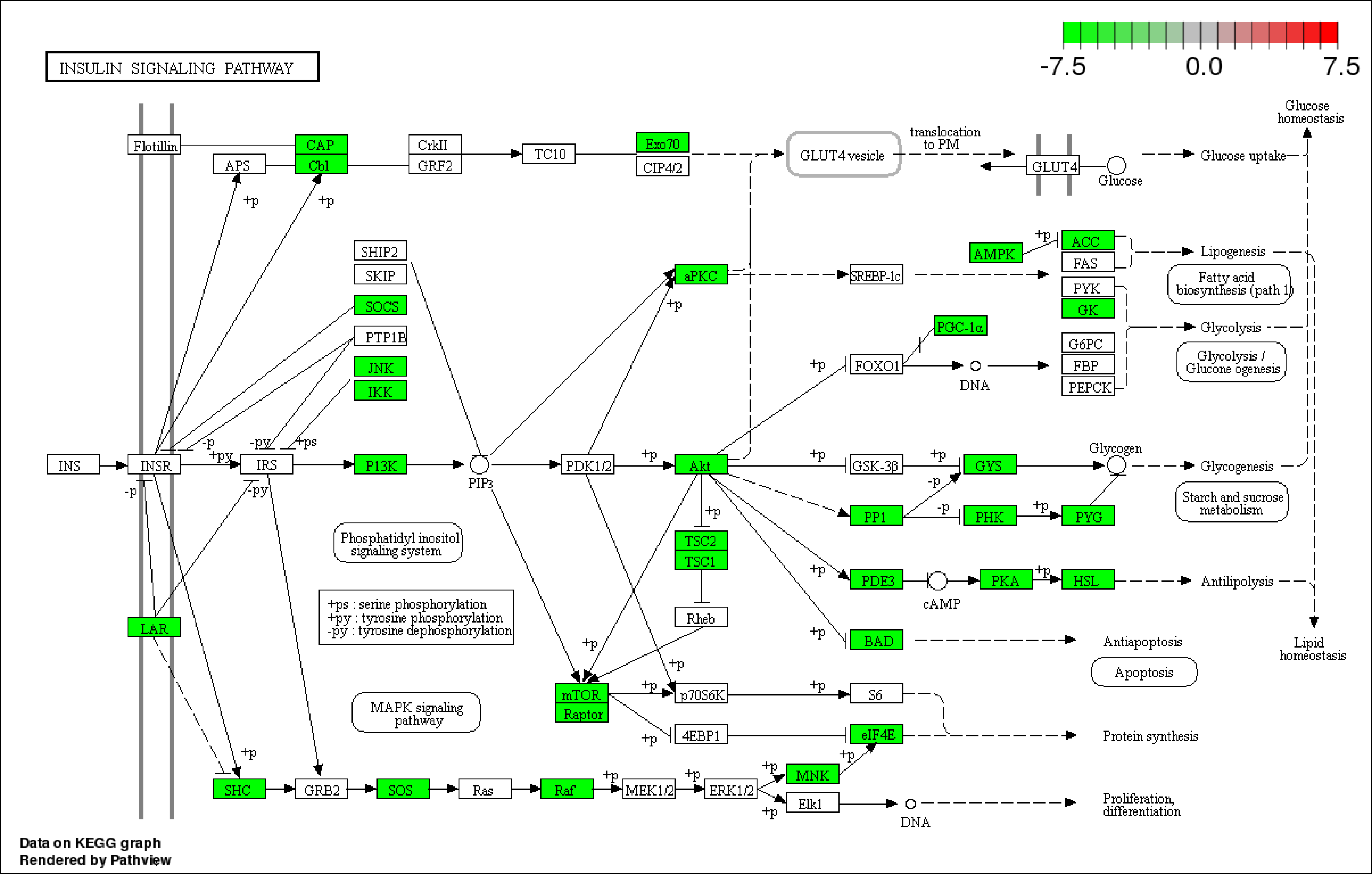

-7.5
0.0
7.5
INSULIN SIGNALING PATHWAY
Flotillin
CAP
Cbl
APS
CrkII
GRF2
TC10
Exo70
CIP4/2
GLUT4 vesicle
translocation to PM
GLUT4
Glucose
Glucose homeostasis
Glucose uptake
SHIP2
SKIP
SOCS
PTP1B
JNK
IKK
aPKC
SREBP-1c
AMPK
ACC
FAS
Lipogenesis
Fatty acid biosynthesis (path 1)
PYK
GK
Glycolysis
PGC-1α
FOXO1
DNA
G6PC
FBP
PEPCK
Glycolysis / Gluconeogenesis
INS
INSR
IRS
PI3K
PIP3
PDK1/2
Akt
GSK-3β
GYS
Glycogen
Glycogenesis
PP1
PHK
PYG
Starch and sucrose metabolism
TSC2
TSC1
Rheb
PDE3
cAMP
PKA
HSL
Antilipolysis
Lipid homeostasis
BAD
Antiapoptosis
Apoptosis
LAR
Phosphatidyl inositol signaling system
+ps : serine phosphorylation
+py : tyrosine phosphorylation
-py : tyrosine dephosphorylation
mTOR
Raptor
p70S6K
S6
4EBP1
eIF4E
Protein synthesis
MAPK signaling pathway
SHC
GRB2
SOS
Ras
Raf
MEK1/2
ERK1/2
MNK
Elk1
DNA
Proliferation, differentiation
Data on KEGG graph
Rendered by Pathview

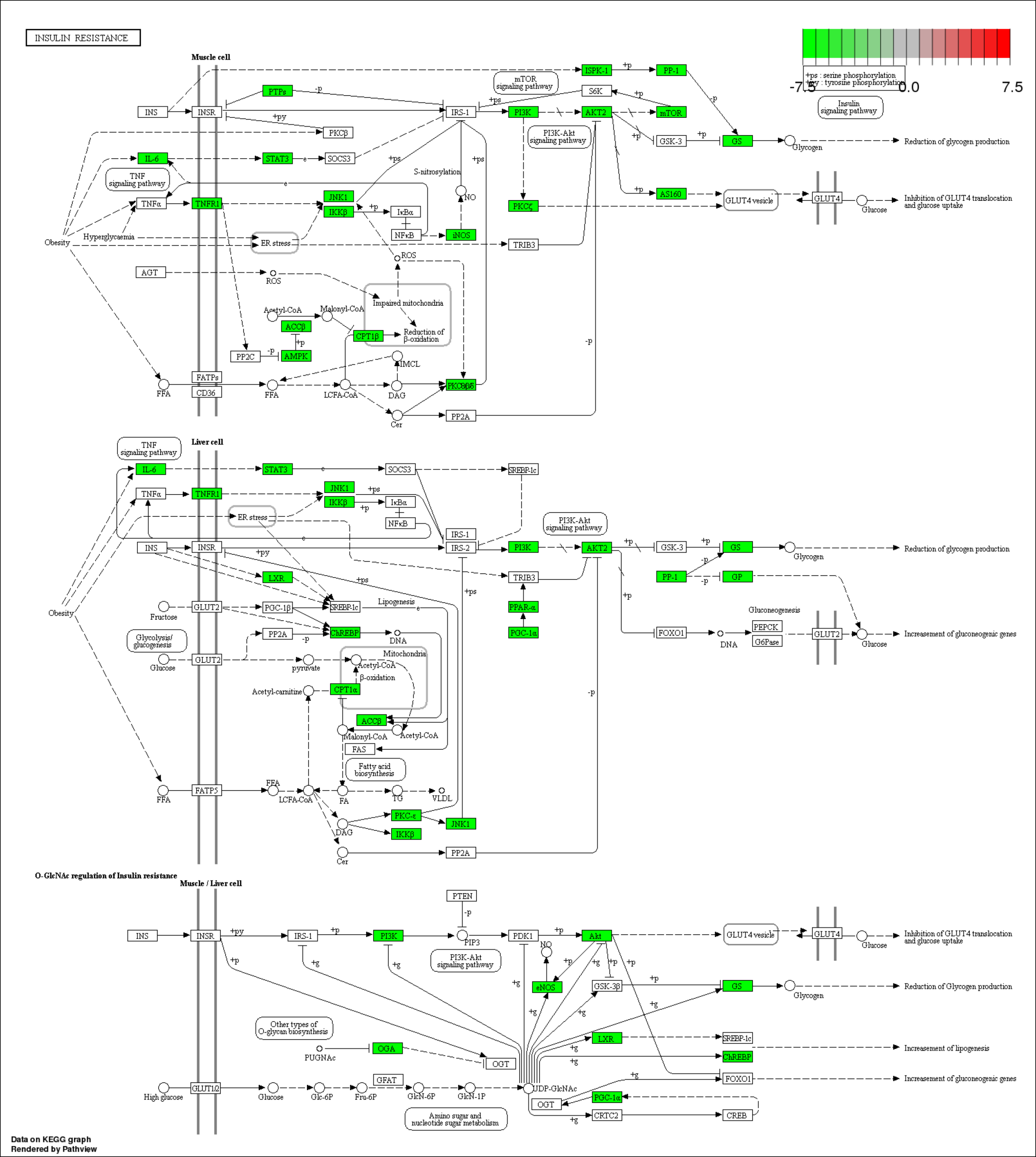
INSULIN RESISTANCE
-7.5
0.0
7.5
+ps : serine phosphorylation
+py : tyrosine phosphorylation
Muscle cell
INS
INSR
PTPs
IRS-1
PI3K
AKT2
mTOR
ISPK-1
PP-1
S6K
GSK-3
GS
Glycogen
Reduction of glycogen production
mTOR signaling pathway
PI3K-Akt signaling pathway
Insulin signaling pathway
PKCβ
IL-6
STAT3
SOCS3
TNF signaling pathway
TNFα
TNFR1
JNK1
IKKβ
IκBα
NFκB
iNOS
NO
S-nitrosylation
PKCζ
AS160
GLUT4 vesicle
GLUT4
Glucose
Inhibition of GLUT4 translocation and glucose uptake
Obesity
Hyperglycaemia
ER stress
TRIB3
AGT
ROS
Impaired mitochondria
Acetyl-CoA
Malonyl-CoA
ACCβ
CPT1β
Reduction of β-oxidation
PP2C
AMPK
IMCL
FATPs
CD36
FFA
LCFA-CoA
DAG
PKCθ/δ/ε
Cer
PP2A
Liver cell
SREBP-1c
IRS-2
GP
FOXO1
DNA
PEPCK
G6Pase
GLUT2
Gluconeogenesis
Increasement of gluconeogenic genes
PPAR-α
PGC-1α
LXR
Fructose
PGC-1β
Lipogenesis
ChREBP
Glycolysis/ glucogenesis
pyruvate
Mitochondria
β-oxidation
Acetyl-carnitine
CPT1α
FAS
Fatty acid biosynthesis
FATP5
FA
TG
VLDL
PKC-ε
O-GlcNAc regulation of Insulin resistance
Muscle / Liver cell
PTEN
PIP3
PDK1
Akt
eNOS
GSK-3β
Reduction of Glycogen production
Other types of O-glycan biosynthesis
PUGNAc
OGA
OGT
Increasement of lipogenesis
High glucose
GLUT1/2
Glc-6P
Fru-6P
GFAT
GlcN-6P
GlcN-1P
UDP-GlcNAc
CRTC2
CREB
Amino sugar and nucleotide sugar metabolism
Data on KEGG graph
Rendered by Pathview

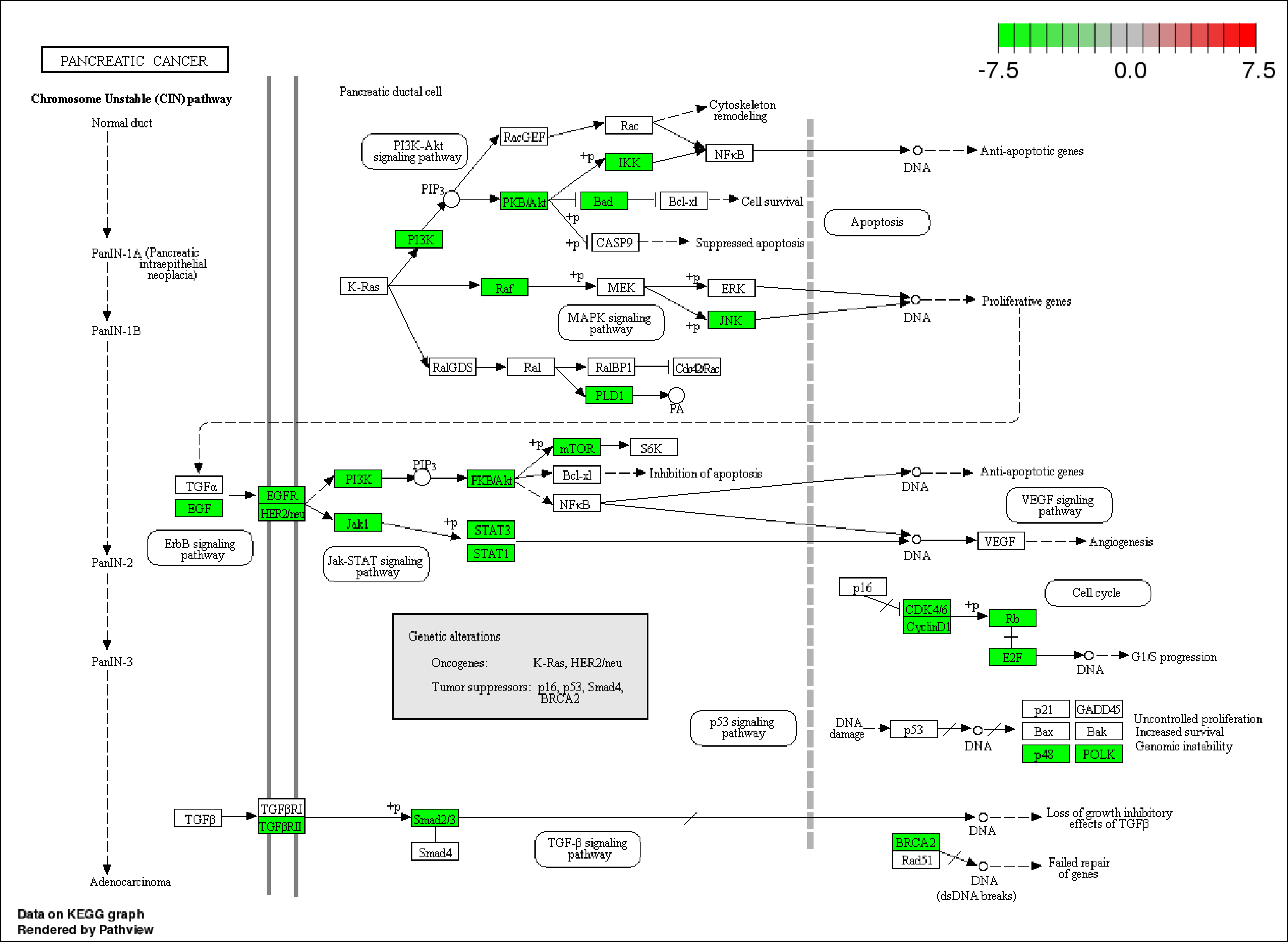

-7.5
0.0
7.5
PANCREATIC CANCER
Chromosome Unstable (CIN) pathway
Normal duct
PanIN-1A (Pancreatic intraepithelial neoplacia)
PanIN-1B
PanIN-2
PanIN-3
Adenocarcinoma
Pancreatic ductal cell
PI3K-Akt signaling pathway
RacGEF
Rac
Cytoskeleton remodeling
IKK
NFκB
Anti-apoptotic genes
DNA
PIP3
PKB/Akt
Bad
Bcl-xl
Cell survival
PI3K
CASP9
Suppressed apoptosis
Apoptosis
K-Ras
Raf
MEK
ERK
JNK
Proliferative genes
MAPK signaling pathway
RalGDS
Ral
RalBP1
Cdc42/Rac
PLD1
PA
mTOR
S6K
Inhibition of apoptosis
TGFα
EGF
EGFR
HER2/neu
Jak1
STAT3
STAT1
VEGF signling pathway
VEGF
Angiogenesis
ErbB signaling pathway
Jak-STAT signaling pathway
p16
CDK4/6
CyclinD1
Rb
E2F
Cell cycle
G1/S progression
Genetic alterations
Oncogenes: K-Ras, HER2/neu
Tumor suppressors: p16, p53, Smad4, BRCA2
p53 signaling pathway
DNA damage
p53
p21
GADD45
Bax
Bak
p48
POLK
Uncontrolled proliferation
Increased survival
Genomic instability
TGFβ
TGFβRI
TGFβRII
Smad2/3
Smad4
TGF-β signaling pathway
Loss of growth inhibitory effects of TGFβ
BRCA2
Rad51
Failed repair of genes
DNA (dsDNA breaks)
Data on KEGG graph
Rendered by Pathview
