# Supplementary figures and images for: In silico predictions of protein interactions between Zika virus and human host
Source: PeerJ. 2021 Aug 24;9:e11770. doi: 10.7717/peerj.11770 (PMC8395582; doi:10.7717/peerj.11770)

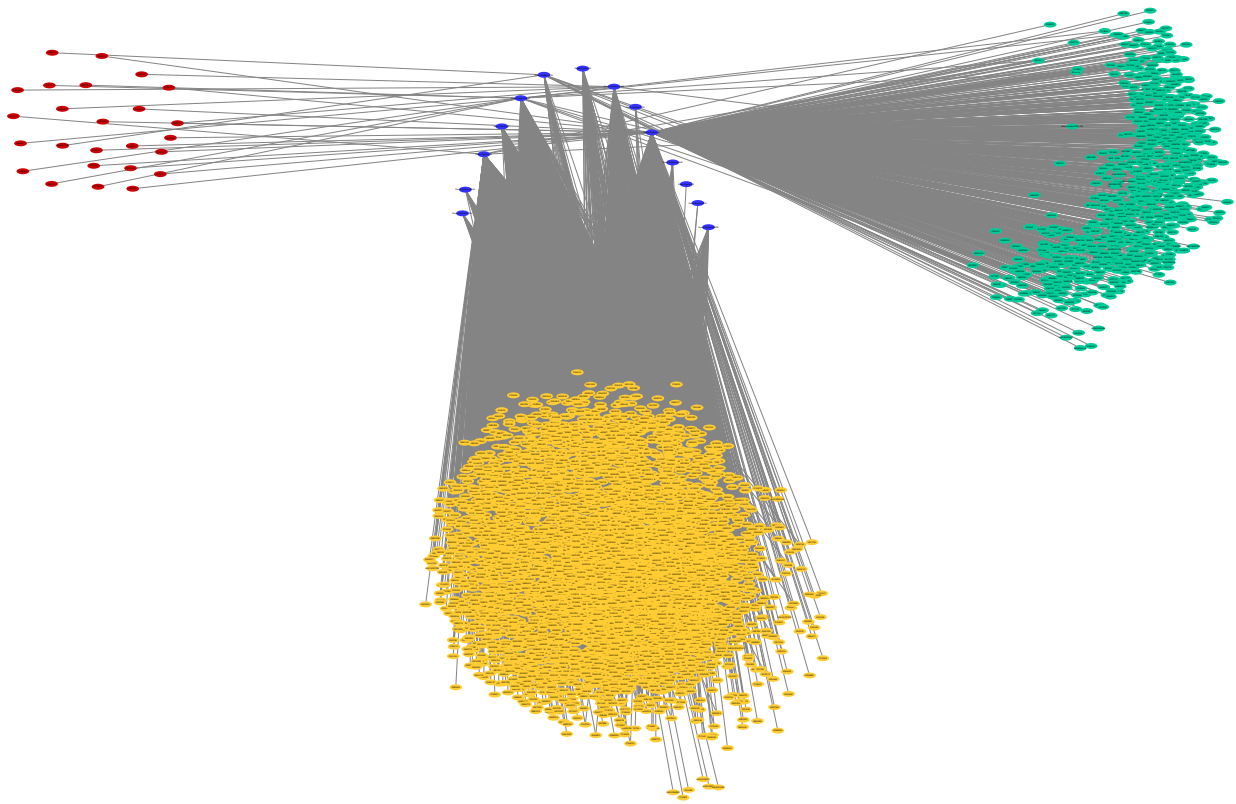

Supplement: Supplemental Information 6 — The blue nodes in the middle represent ZIKV collapsed proteins from the two ZIKV strains analyzed (FSS13025 and PE243). The yellow nodes are human proteins that interact with both FSS13025 and PE243 strains. Red nodes are human proteins that interact only with the FSS13025 strain and green nodes are human proteins that interact only with the PE243 strain. [file peerj-09-11770-s006.pdf]
